# Association between air pollution and lifestyle with the risk of developing mild cognitive impairment and dementia in individuals with cardiometabolic diseases

**DOI:** 10.1038/s41598-024-83607-w

**Published:** 2025-01-15

**Authors:** Bo Wang, Lingling Yang, Ting Ma, Shulan He, Jiangping Li, Xian Sun

**Affiliations:** 1 School of Public Health, Ningxia Medical University, Yinchuan, 750004 Ningxia China; 2 Key Laboratory of Environmental Factors and Chronic Disease Control, No.1160, Shengli Street, Xingqing District, Yinchuan, 750004 Ningxia China

**Keywords:** Ambient air pollution, Lifestyle, Mild cognitive impairment, Dementia, Cardiometabolic diseases, Epidemiology, Neurological disorders

## Abstract

Lifestyle factors and ambient air pollution are linked to dementia and CMDs, yet few studies have investigated their impact on dementia risk in CMDs patients at the same time. The Cox proportional hazards model was used to evaluate the influence of lifestyle and ambient air pollution on the dementia risk of the CMDs population among 438,681 participants in the UK Biobank. It is found that the risk of developing mild cognitive impairment and dementia in the population seems to increase with the increase in the number of CMDs. There appears to be a statistically significant association between high levels of ambient air pollution, unhealthy lifestyles, and a higher risk of developing mild cognitive impairment and dementia in the CMDs population. It is found that a healthy lifestyle may have an effect modifier role in the association between ambient air pollution and the risk of mild cognitive impairment and the development of dementia in patients with CMDs. Therefore, maybe people with CMDs can lessen the impact of ambient air pollution on their risk of developing mild cognitive impairment and dementia by improving their lifestyle.

**Supplementary Information:**

The online version contains supplementary material available at 10.1038/s41598-024-83607-w.

## Introduction

In just 26 years, from 1990 to 2016, the global number of people with dementia increased by a factor of 1.17 due to aging and population growth^1^. There are currently about 57.4 million individuals worldwide with dementia^2^, leading to economic losses of $1,313.4 billion^3^. Dementia has emerged as a significant barrier to healthy aging in contemporary society. As an irreversible neurodegenerative condition, dementia still lacks an effective treatment. Thus, preventing dementia through managing risk factors and creating practical interventions continues to be a significant public health challenge.

Cardiometabolic diseases (CMDs), including type 2 diabetes mellitus (T2DM), stroke, and coronary heart disease (CHD)^4^, are well-documented independent risk factors for cognitive impairment and dementia^5^, each of which is associated with a 2-fold increased risk of dementia^6–8^. One-third of older adults globally have at least two comorbidities of CMDs^9^. It is known that CMDs can act in synergy to increase the risk of adverse health outcomes^10^. CMDs multimorbidity speeds up cognitive decline and raises the chance of transitioning to dementia in individuals without dementia^9^. Previous studies have shown that the risk of cognitive impairment and dementia in the population can be reduced by preventing or delaying the onset and development of CMDs such as T2DM^11^, stroke^12^, and CHD^13^. In addition, the prevalence of CMDs is increasing rapidly^4^, and there exists a dose-dependent correlation between CMDs and the occurrence and development of dementia^9^. Therefore, a new direction of research with high health benefits to reduce the risk of dementia is to explore measures to delay or prevent the onset and development of CMDs.

Pharmacological treatments for dementia are currently limited in value, making a shift to primary prevention measures a top priority. Previous studies have shown that exposure to ambient air pollutants^14,15^, along with unhealthy habits such as smoking^16,17^, alcohol abuse^17,18^, and low physical activity^17,19^, are the same risk factors for developing both dementia and CMDs. Evidence shows that primary prevention measures such as reducing exposure to ambient air pollution and adopting a healthy lifestyle can reduce the risk of dementia^14,20^ and CMDs in the general population^21,22^. However, it remains unclear whether this primary prevention could be extended to CMDs patients to delay disease progression, reduce disease burden, and reduce the risk of complications such as dementia. In addition, ambient air pollutants enter the brain through the bloodstream, destroying the blood-brain barrier, activating microglia, and releasing many pro-inflammatory mediators, triggering a neuroimmune response^23^. This leads to the aggravation of inflammatory responses and oxidative stress in the body^24^, promotes the development of metabolic dysfunction diseases, and increases the risk of cardiovascular metabolic diseases, such as stroke^23^, thus further increasing the risk of dementia^25,26^. Maintaining a healthy lifestyle can help reduce inflammation, inhibit oxidative stress^27^, and also help prevent cognitive impairment caused by CMDs^28^. However, it is unclear whether the risk of cognitive impairment and dementia caused by ambient air pollution can be further reduced in people with cardiometabolic disease by adopting a healthy lifestyle. Previous studies have not determined whether and to what extent exposure to ambient air pollution increases the risk of cardiometabolic disease-related dementia, and whether and to what extent this risk can be offset by a broad combination of healthy lifestyle factors. Understanding these characteristics can help develop future dementia prevention interventions for at-risk populations.

To our knowledge, few studies have explored the link between ambient air pollution and the risk of developing dementia in cardiometabolic disease patients, and the potential impact of a healthy lifestyle. To fill these gaps, the present study investigated the influence of ambient air pollution and a healthy lifestyle on the risk of dementia development in patients with cardiometabolic diseases and the potential impact of a healthy lifestyle in a large prospective cohort study. The results were compared with those of the population without CMDs to provide theoretical support for the improvement of clinical symptoms in patients with cardiometabolic disease and the possible delay or prevention of the onset of cognitive impairment and dementia in the population.

## Results

### Baseline characteristics of the participants

Table 1 depicts the participant characteristics. Out of 438,681 participants, 75,056 (17.11%) had CMDs. Among them, 15.12% had one CMDs, 1.90% had two CMDs, and 0.09% had three CMDs. Individuals with one, two, or three CMDs were compared to those without CMDs. The former group was older, had more males and retirees, lower education levels, poorer economic status, lower normal BMI rates, fewer carriers of the APOE ε4 gene, more dyslipidemia and hypertriglyceridemia, and higher usage of lipid-lowering drugs and aspirin. Furthermore, this group has a high proportion of patients with moderate and severe serum 25(OH)D deficiency and hypertension, and a relatively low proportion of patients with depression. Significant statistical differences in exposure to ambient air pollutants and healthy lifestyle scores were found between people with and without CMDs, with a higher proportion of people with CMDs being exposed to medium and high levels of ambient air pollutants compared to those without CMDs. Healthy lifestyle scores of 0–1, 2–3, and 4 were higher in individuals with CMDs compared to those without CMDs, whereas the proportion of scores of 5–7 was lower in those with CMDs.

**Table 1:** Characteristics of participants with or without Cardiometabolic diseases.

Characteristic	Total population (*N* = 438681)	Number of CMDs			
		No(*N* = 363625)	One(*N* = 66324)	Two(*N* = 8318)	Three(*N* = 414)
Age, years, mean (SD)	56.51(8.08)	55.73(8.10)	60.04(6.97)	61.71(6.06)	62.24(5.69)
Sex, n(%)					
Female	239,091(54.50)	210,973(58.02)	25,579(38.57)	2435(29.27)	104(25.12)
Male	199,590(45.50)	152,652(41.98)	40,745(61.43)	5883(70.73)	310(74.88)
Race/ethnicity, n(%)					
White	414,781(94.55)	344,941(94.86)	62,036(93.53)	7438(89.42)	366(88.41)
Asian	9352(2.13)	6768(1.86)	2050(3.09)	509(6.12)	25(6.04)
Black	6794(1,55)	5559(1.53)	1059(1.60)	169(2.03)	7(1.69)
Other	6354(1.45)	5234(1.44)	945(1.42)	161(1.94)	14(3.38)
Missing	1400(0.32)	1123(0.31)	234(0.35)	41(0.49)	2(0.48)
Occupation status, n(%)					
Employed	257,444(58.69)	226,329(62.24)	28,551(43.05)	2505(30.12)	59(14.25)
Unemployed	31,835(7.26)	24,424(6.72)	6016(9.07)	1289(15.50)	106(25.60)
Retired	145,660(33.20)	109,807(30.20)	31,157(46.98)	4452(53.52)	244(58.94)
Missing	3742(0.85)	3065(0.84)	600(0.90)	72(0.87)	5(1.21)
Education level, n(%)					
College or University	141,860(32.34)	123,544(33.98)	16,623(25.06)	1615(19.42)	78(18.84)
A level/AS level or equivalent	49,209(11.22)	42,384(11.66)	6132(9.25)	673(8.09)	20(4.83)
O level/GCSEs or equivalent	95,155(21.69)	80,020(22.01)	13,444(20.27)	1619(19.46)	72(17.39)
Professional Qualifications	76,016(17.33)	61,687(16.96)	12,637(19.05)	1627(19.56)	65(15.70)
None of the above	69,280(15.79)	50,455(13.88)	16,081(24.25)	2573(30.93)	171(41.30)
Missing	7161(1.63)	5535(1.52)	1407(2.12)	211(2.54)	8(43.24)
TDI, n(%)					
Low	80,275(18.30)	63,050(17.34)	14,565(21.96)	2491(29.95)	169(40.82)
Medium	130,756(29.81)	108,356(29.80)	19,754(29.78)	2532(30.44)	114(27.54)
High	227,151(51.78)	191,803(52.75)	31,927(48.14)	3290(39.55)	131(31.64)
Missing	499(0.11)	416(0.11)	78(0.12)	5(0.06)	0(0.00)
BMI (kg/m2), n(%)					
< 18.5	2272(0.52)	2052(0.56)	200(0.30)	20(0.24)	0(0.00)
18.5–24.9	143,735(32.77)	129,391(35.58)	13,464(20.30)	847(10.18)	33(7.97)
25-29.9	186,225(42.45)	154,297(42.43)	28,811(43.44)	3004(36.11)	113(27.29)
≥ 30	104,323(23.78)	76,336(20.99)	23,388(35.26)	4337(52.14)	262(63.29)
Missing	2126(0.48)	1549(0.43)	461(0.70)	110(1.32)	6(1.45)
APOE Ƹ4 genotype, n(%)					
No	323,909(73.84)	268,410(73.82)	49,023(73.91)	6155(74.00)	321(77.54)
Yes	102,847(23.44)	85,460(23.50)	15,423(23.25)	1883(22.64)	81(19.57)
Missing	11,925(2.72)	9755(2.68)	1878(2.83)	280(3.37)	12(2.90)
Hypertension status, n(%)					
No	268,504(61.21)	239,021(65.73)	27,554(41.54)	1875(22.54)	54(13.04)
Yes	170,177(38.79)	124,604(34.27)	38,770(58.46)	6443(77.46)	360(86.96)
Depressive status, n(%)					
No	333,544(76.03)	274,260(75.42)	52,322(78.89)	6629(79.69)	333(80.43)
Yes	105,137(23.97)	89,365(24.58)	14,002(21.11)	1689(20.31)	81(19.57)
Dyslipidemia, n(%)					
No	364,693(83.13)	308,945(84.96)	50,322(75.87)	5184(62.32)	242(58.45)
Yes	73,988(16.87)	54,680(15.04)	16,002(24.13)	3134(37.68)	172(41.55)
Hypertriglyceridemia, n(%)					
No	275,318(62.76)	235,779(64.84)	35,411(53.39)	3945(47.43)	183(44.20)
Yes	163,363(37.24)	127,846(35.16)	30,913(46.61)	4373(52.57)	231(55.80)
Serum 25(OH)D status, n(%)					
Severely deficient	47,813(10.90)	37,845(10.41)	8407(12.68)	1476(17.74)	85(20.53)
Moderately deficient	162,302(37.00)	133,753(36.78)	25,012(37.71)	3362(40.42)	175(42.27)
Insufficient and above	181,575(41.39)	152,776(42.01)	25,988(39.18)	2700(32.46)	111(26.81)
Missing	46,991(10.71)	39,251(10.79)	6917(10.43)	780(9.38)	43(10.39)
Lipid-lowering medication usage, n(%)					
No	365,619(83.35)	328,091(90.23)	35,740(53.89)	1745(20.98)	43(10.39)
Yes	73,062(16.65)	35,534(9.77)	30,584(46.11)	6573(79.02)	371(89.61)
Aspirin use status, n(%)					
No	380,755(86.80)	335,222(92.19)	42,199(63.63)	3213(38.63)	121(29.23)
Yes	57,926(13.20)	28,403(7.81)	24,125(36.37)	5105(61.37)	293(70.77)
Healthy lifestyle score					
0–1	5673(1.29)	4303(1.18)	1149(1.73)	204(2.45)	17(4.11)
2–3	126,723(28.89)	100,912(27.75)	22,240(33.53)	3370(40.51)	201(48.55)
4	130,806(29.82)	108,162(29.75)	20,032(30.20)	2486(29.89)	126(30.43)
5–7	175,479(40.00)	150,248(41.32)	22,903(34.53)	2258(27.15)	70(16.91)
Ambient air pollution, n(%)					
Low	136,374(31.09)	114,967(31.62)	19,358(29.19)	1965(23.62)	84(20.29)
Medium	158,524(36.14)	130,936(36.01)	24,318(36.67)	3122(37.53)	148(35.75)
High	143,783(32.78)	117,722(32.37)	22,648(34.15)	3231(38.84)	182(43.96)
PM_2.5_ (µg/m^3^), n(%)					
Q1	111,914(25.51)	94,394(25.96)	15,840(23.88)	1614(19.40)	66(15.94)
Q2	109,733(25.01)	91,185(25.08)	16,447(24.80)	2004(24.09)	97(23.43)
Q3	109,104(24.87)	89,860(24.71)	16,882(25.45)	2256(27.12)	106(25.60)
Q4	107,930(24.60)	88,186(24.25)	17,155(25.87)	2444(29.38)	145(35.02)
PM_2.5–10_ (µg/m^3^), n(%)					
Q1	111,959(25.52)	93,549(25.73)	16,383(24.70)	1929(23.19)	98(23.67)
Q2	110,324(25.15)	91,559(25.18)	16,574(24.99)	2092(25.15)	99(23.91)
Q3	108,160(24.66)	89,409(24.59)	16,545(24.95)	2103(25.28)	103(24.88)
Q4	108,238(24.67)	89,108(24.51)	16,822(25.36)	2194(26.38)	114(27.54)
PM_10_ (µg/m^3^), n(%)					
Q1	110,942(25.29)	92,804(25.52)	16,266(24.53)	1782(21.42)	90(21.74)
Q2	110,594(25.21)	91,756(25.23)	16,618(25.06)	2109(25.35)	111(26.81)
Q3	108,544(24.74)	89,552(24.63)	16,742(25.24)	2162(25.99)	88(21.26)
Q4	108,601(24.76)	89,513(24.62)	16,698(25.18)	2265(27.23)	125(30.19)
NO_2_ (µg/m^3^), n(%)					
Q1	111,556(25.43)	94,241(25.92)	15,706(23.68)	1543(18.55)	66(15.94)
Q2	110,210(25.12)	91,150(25.07)	16,870(25.44)	2086(25.08)	104(25.12)
Q3	109,214(24.90)	89,746(24.68)	17,070(25.74)	2285(27.47)	113(27.29)
Q4	107,701(24.55)	88,488(24.33)	16,678(25.15)	2404(28.90)	131(31.64)
NO_X_ (µg/m^3^), n(%)					
Q1	111,333(25.38)	94,153(25.89)	15,573(23.48)	1539(18.50)	68(16.43)
Q2	110,246(25.13)	91,371(25.13)	16,695(25.17)	2089(25.11)	91(21.98)
Q3	109,145(24.88)	89,711(24.67)	17,075(25.74)	2248(27.03)	111(26.81)
Q4	107,957(24.61)	88,390(24.31)	16,981(25.60)	2442(29.36)	144(34.78)
Mild cognitive impairment, n(CIR)	279(0.043)	203(0.038)	62(0.060)	14(0.091)	0(0.000)
All-cause dementia, n(CIR)	8101(1.243)	5032(0.943)	2463(2.407)	557(3.649)	49(6.449)
Alzheimer’s disease, n(CIR)	3635(0.556)	2433(0.455)	994(0.967)	193(1.253)	15(1.885)
Vascular dementia, n(CIR)	1737(0.266)	830(0.155)	668(0.649)	209(1.353)	30(3.814)
Survival time, M (P25, P75), year					
Cognitive impairment	14.91(14.30,15.74)	14.71(14.33,15.68)	15.53(14.09,16.10)	18.49(12.72,22.51)	18.65(10.12,24.60)
All-cause dementia	14.86(14.23,15.73)	14.68(14.31,15.68)	15.43(14.04,16.08)	18.35(12.52,22.26)	18.35(9.83,24.27)
Alzheimer’s disease	14.90(14.29,15.73)	14.70(14.32,15.68)	15.51(14.07,16.10)	18.51(12.78,22.52)	19.22(10.12,24.77)
Vascular dementia	14.91(14.30,15.74)	14.71(14.33,15.68)	15.52(14.08,16.10)	18.56(12.90,22.60)	19.00(10.63,24.52)

Note: Data is n (%).

Abbreviations: mean (SD), arithmetic mean (Standard Deviation); TDI, Townsend deprivation index; BMI, Body mass index; APOE, apolipoprotein E; PM_2.5_, particulate matter with aerodynamic diameter ≤ 2.5 μm; PM_10_, particulate matter with an aerodynamic diameter ≤ 10 μm; PM_2.5–10_, particulate matter with an aerodynamic diameter between 2.5 and 10 μm; NO_2_, nitrogen dioxide; NO_X_, nitrogen oxides; Q 1: quartile 1; CIR: Crude incidence rate; M (P_25_, P_75_), median (Percentile_25_, Percentile_75_).

The risk of developing mild cognitive impairment, all-cause dementia, Alzheimer’s disease, and vascular dementia in patients with CMDs was 1.951, 1.554, 1.216, and 2.032 times higher than in those without CMDs, respectively [mild cognitive impairment: *HR = 1.951*,* 95% CI: 1.404*,* 2.710*; all-cause dementia: *HR = 1.554*,* 95% CI: 1.473*,* 1.640*; Alzheimer’s disease: *HR = 1.216*,* 95% CI: 1.204*,* 1.228*; vascular dementia: *HR = 2.032*,* 95% CI: 1.799*,* 2.296*]. The risk of developing mild cognitive impairment, all-cause dementia, Alzheimer’s disease, and vascular dementia in the population seems to increase with the increase in the number of CMDs. Patients with all three types of CMDs have the highest risk of total dementia, Alzheimer’s disease, and vascular dementia (Table 2). Furthermore, the rates of mild cognitive impairment, all-cause dementia, Alzheimer’s disease, and vascular dementia per 1,000 person-years were 0.038 (95% CI: 0.03, 0.04), 0.943 (95% CI: 0.92, 0.97), 0.455 (95% CI: 0.44, 0.47) and 0.155 (95% CI: 0.14, 0.17), respectively, for those without CMDs. The prevalence rates of mild cognitive impairment, all-cause dementia, Alzheimer’s disease, and vascular dementia per 1000 person-years in the CMDs population were 0.064 (95% CI: 0.05, 0.08), 2.593 (95% CI: 2.50, 2.69) and 1.010 (95% CI: 0.95, 1.010), respectively (Table 3).

**Table 2:** Cardiometabolic disease status and risk of cognitive impairment and different subtypes of dementia in the population: hazard ratios with 95% CI.

CMDs status	Adjust HR(95%CI)			
	Mild Cognitive impairment	All-cause dementia	Alzheimer’s disease	Vascular dementia
No	1(Ref)	1(Ref)	1(Ref)	1(Ref)
Yes	1.951(1.404,2.710)	1.554(1.473,1.640)	1.216(1.204,1.228)	2.032(1.799,2.296)
Only one	1.859(1.324,2.610)	1.510(1.428,1.597)	1.193(1.096,1.298)	2.039(1.811,2.297)
Diabetes	0.969(0.436,2.158)	1.116(0.995,1.251)	0.759(0.630,0.914)	1.319(1.054,1.651)
Stroke	0.812(0.193,3.415)	1.419(1.199,1.680)	0.600(0.439,0.821)	2.337(1.774,3.079)
CHD	2.213(1.554,3.152)	1.626(1.532,1.727)	1.379(1.260,1.509)	2.224(1.974,2.550)
Any two	3.230(1.686,6.184)	1.841(1.658,2.045)	1.120(0.941,1.333)	2.492(2.058,3.018)
Diabetes & Stroke	0.000(0.000,2.983^92)	1.352(0.848,2.157)	0.311(0.099,0.972)	2.128(1.088,4.166)
Diabetes & CHD	3.541(1.745,7.186)	1.762(1.559,1.992)	1.095(0.894,1.341)	2.283(1.823,2.858)
CHD & Stroke	1.836(0.433,7.783)	1.887(1.570,2.269)	0.730(0.513,1.040)	2.533(1.852,3.464)
Three (Diabetes & CHD & Stroke)	0.000(0.000,7.744^86)	3.426(2.513,4.669)	1.599(0.899,2.844)	6.632(4.368,10.069)
*P* value	< 0.001	< 0.001	< 0.001	< 0.001

Abbreviations: CMDs, Cardiometabolic diseases; CHD, coronary heart disease; HR, hazard ratios; CI, Confidence Intervals.

In the model, we controlled for basic sociodemographic factors [age, sex, race, educational level, occupational status, TDI, BMI] and health-related concerns [APOE genotype; history of hypertension; history of depression; dyslipidemia; hypertriglyceridemia; aspirin use; lipid-lowering medication use; serum 25(OH)D levels].

**Table 3:** Cardiometabolic Disease Status and Prevalence of Cognitive Impairment and Various Dementia Subtypes in Populations: per 1000 person-year.

CMDs status	Mild cognitive impairment			All-cause dementia			Alzheimer’s disease			Vascular dementia		
	Events	Person-years	per 1000 person-year	Events	Person-years	per 1000 person-year	Events	Person-years	per 1000 person-year	Events	Person-years	per 1000 person-year
No	203	5,350,681	0.038(0.03,0.04)	5032	5,336,325	0.943(0.92,0.97)	2433	5,344,792	0.455(0.44,0.47)	830	5,349,658	0.155(0.14,0.17)
Yes	76	1,191,792	0.064(0.05,0.08)	3069	1,183,381	2.593(2.50,2.69)	1202	1,190,387	1.010(0.95,1.07)	907	1,191,859	0.761(0.71,0.81)
Only one	62	1,030,236	0.060(0.05,0.08)	2463	1,023,120	2.407(2.31,2.50)	994	1,028,430	0.967(0.91,1.03)	668	1,029,533	0.649(0.60,0.70)
Diabetes	7	279,253	0.025(0.01,0.05)	410	278,215	1.474(1.34,1.63)	175	279,260	0.627(0.54,0.73)	118	279,416	0.422(0.35,0.51)
Stroke	2	84,227	0.024(0.00,0.10)	178	83,806	2.124(1.83,2.47)	54	84,358	0.640(0.49,0.84)	80	84,480	0.947(0.76,1.18)
CHD	53	666,754	0.079(0.06,0.10)	1875	661,099	2.836(2.71,2.97)	765	664,811	1.151(1.07,1.24)	470	665,636	0.706(0.64,0.77)
Any two	14	153,836	0.091(0.05,0.16)	557	152,662	3.649(3.36,3.97)	193	153,999	1.253(1.09,1.45)	209	154,459	1.353(1.18,1.55)
Diabetes & Stroke	0	8012	0.000(0.00,0.60)	22	7943	2.770(1.78,4.27)	5	8014	0.624(0.23,1.55)	12	8142	1.474(0.80,2.65)
Diabetes & CHD	11	109,101	0.101(0.05,0.19)	377	108,349	3.479(3.14,3.85)	146	109,185	1.337(1.13,1.58)	130	109,421	1.188(1.00,1.47)
CHD & Stroke	3	336,721	0.009(0.00,0.03)	158	36,369	4.344(3.71,5.09)	42	36,799	1.141(0.83,1.56)	67	36,896	1.816(1.42,2.32)
Three(Diabetes & CHD & Stroke)	0	7719	0.000(0.00,0.62)	49	7598	6.449(4.83,8.59)	15	7957	1.885(1.10,3.19)	30	7866	3.814(2.62,5.51)

Abbreviations: CMDs, Cardiometabolic disease; CHD, coronary heart disease.

### Effect of ambient air pollution on mild cognitive impairment and dementia risk in individuals with cardiometabolic diseases

**Fig. 1:**
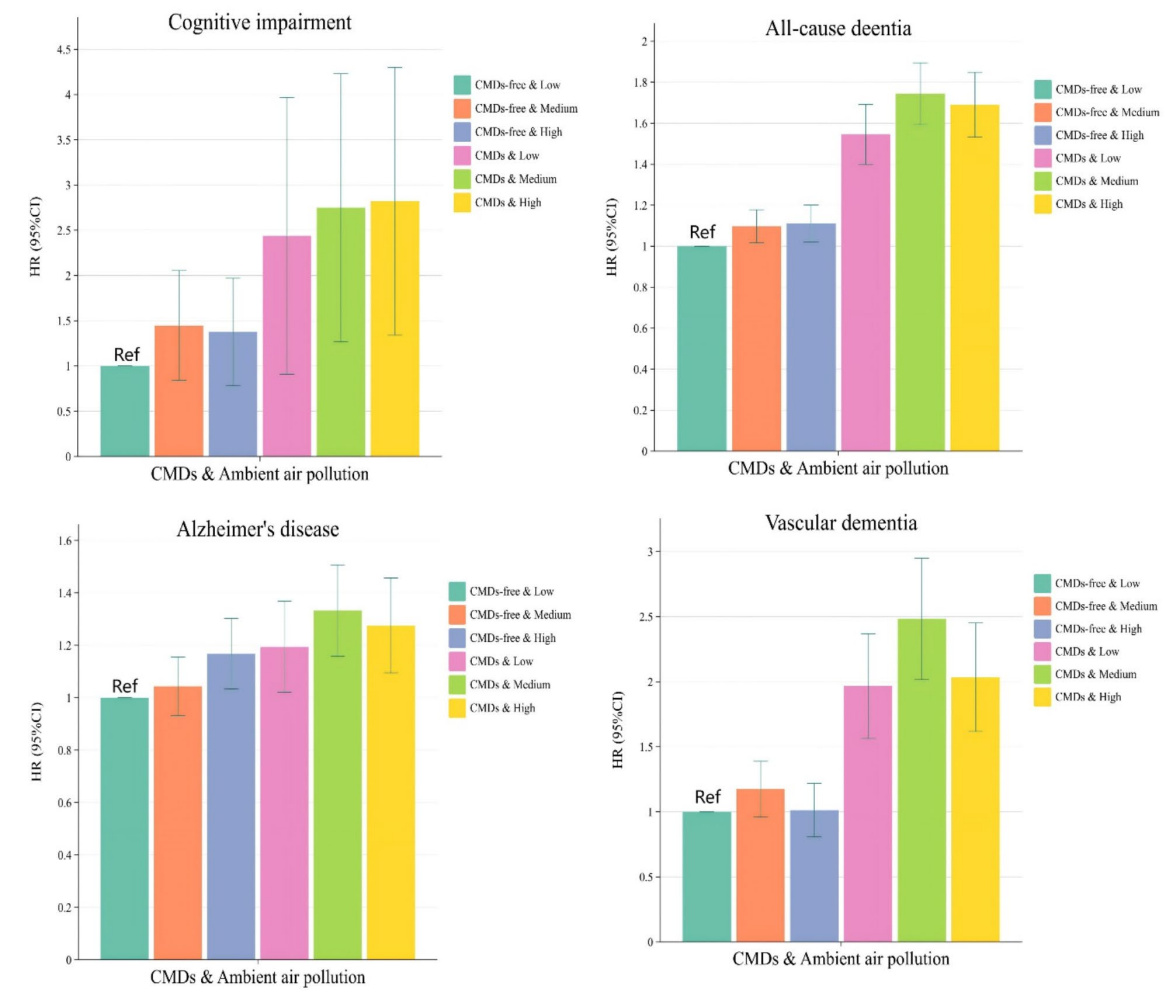
Association between exposure to ambient air pollution and risk of developing mild cognitive impairment and several dementia subtypes in patients with cardiometabolic disease. Abbreviations: CMDs, Cardiometabolic disease; HR, hazard ratios; CI, Confidence Intervals;In the model, we controlled for basic sociodemographic factors [age, sex, race, educational level, occupational status, TDI, BMI] and health-related concerns [APOE genotype; history of hypertension; history of depression; dyslipidemia; hypertriglyceridemia; aspirin use; lipid-lowering medication use; serum 25(OH)D levels].

When analyzed in combination with CMDs status and ambient air pollution factors, patients with non-CMDs and those with CMDs showed consistent associations with a higher risk of mild cognitive impairment, all-cause dementia, Alzheimer’s disease, and vascular dementia if they maintained high levels of ambient air pollution exposure (Fig. 1). The risk of mild cognitive impairment, all-cause dementia, Alzheimer’s disease, and vascular dementia is 2.562 times, 1.686 times, 1.267 times, and 2.006 times higher, respectively, in patients with CMDs and high exposure to ambient air pollution than in patients without CMDs and low exposure to ambient air pollution (Table 4). Among them, high levels of exposure to PM_2.5_, PM_2.5−10_, PM_10_, NO_2_, and NO_X_ may elevate the risk of different dementia subtypes in patients with CMDs (Tables S7). The incidence rates of mild cognitive impairment, all-cause dementia, Alzheimer’s disease, and vascular dementia per 1000 person-years in individuals with CMDs exposed to high levels of ambient air pollution were 0.087 (95% CI: 0.06, 0.12), 2.699 (95% CI: 2.54, 2.86), 1.050 (95% CI: 0.95, 1.15), and 0.743 (95% CI: 0.66, 0.83), respectively (Tables S8).

**Table 4:** Correlation between CMDs status and ambient air pollution combined variables and cognitive impairment and different subtypes of dementia.

CMDs status	Ambient air pollution	Adjust HR (95%CI)			
		Mild cognitive impairment	All-cause dementia	Alzheimer’s disease	Vascular dementia
CMDs-free	Low	1(Ref)	1(Ref)	1(Ref)	1(Ref)
	Medium	1.363(0.888,2.091)	1.095(1.017,1.179)	1.040(0.934,1.157)	1.162(0.969,1.394)
	High	1.290(0.830,2.006)	1.109(1.022,1.202)	1.163(1.036,1.305)	0.999(0.815,1.225)
CMDs	Low	2.117(1.093,4.102)	1.541(1.402,1.694)	1.186(1.025,1.372)	1.939(1.579,2.381)
	Medium	2.485(1.421,4.344)	1.740(1.596,1.897)	1.325(1.162,1.511)	2.455(2.034,2.961)
	High	2.562(1.487,4.413)	1.686(1.536,1.851)	1.267(1.099,1.461)	2.006(1.633,2.465)
	P value	0.003	< 0.001	< 0.001	< 0.001

Abbreviations: CMDs, Cardiometabolic disease; HR, hazard ratios; CI, Confidence Intervals.

In the model, we controlled for basic sociodemographic factors [age, sex, race, educational level, occupational status, TDI, BMI] and health-related concerns [APOE genotype; history of hypertension; history of depression; dyslipidemia; hypertriglyceridemia; aspirin use; lipid-lowering medication use; serum 25(OH)D levels].

In the stratified study using the presence of CMDs, high levels of ambient air pollution increased the risk of all-cause dementia and Alzheimer’s disease in the non-CMDs population by 12.0% and 25.3%, respectively [all-cause dementia: *HR = 1.120*,* 95% CI:1.030*,*1.218*; Alzheimer’s disease: *HR = 1.253*,* 95% CI:1.123*,*1.398*] (Table 5). Compared with the lowest quartile of PM_2.5_ exposure, the risk of all-cause dementia and Alzheimer’s disease in non-CMDs individuals exposed to the highest quartile of PM_2.5_ is 1.137 and 1.242 times higher, respectively (Tables S9). High ambient air pollution exposure increased the risk of developing vascular dementia in patients with CMDs by 1.086 times compared to low exposure (Table 5). In addition, ambient air pollution was not found to be associated with the risk of developing mild cognitive impairment (*P* > 0.05) (Table 5 and Tables S9).

**Table 5:** The influence of ambient air pollution on cognitive impairment and dementia risk of CMDs population and non-CMDs population, stratified by CMDs status.

CMDs status	Ambient air pollution	Adjust HR (95%CI)			
		Mild cognitive impairment	All-cause dementia	Alzheimer’s disease	Vascular dementia
CMDs-free	Low	1(Ref)	1(Ref)	1(Ref)	1(Ref)
	Medium	-	1.097(1.018,1.182)	1.057(0.950,1.157)	-
	High	-	1.120(1.030,1.218)	1.253(1.123,1.398)	-
	P value	0.339	0.014	< 0.001	0.127
CMDs	Low	1(Ref)	1(Ref)	1(Ref)	1(Ref)
	Medium	-	-	-	1.283(1.073,1.535)
	High	-	-	-	1.086(0.897,1.315)
	P value	0.829	0.074	0.372	0.015

Abbreviations: CMDs, Cardiometabolic disease; HR, hazard ratios; CI, Confidence Intervals.

In the model, we controlled for basic sociodemographic factors [age, sex, race, educational level, occupational status, TDI, BMI] and health-related concerns [APOE genotype; history of hypertension; history of depression; dyslipidemia; hypertriglyceridemia; aspirin use; lipid-lowering medication use; serum 25(OH)D levels].

### Effect of healthy lifestyle score on mild cognitive impairment and dementia risk in individuals with cardiometabolic diseases

**Fig. 2:**
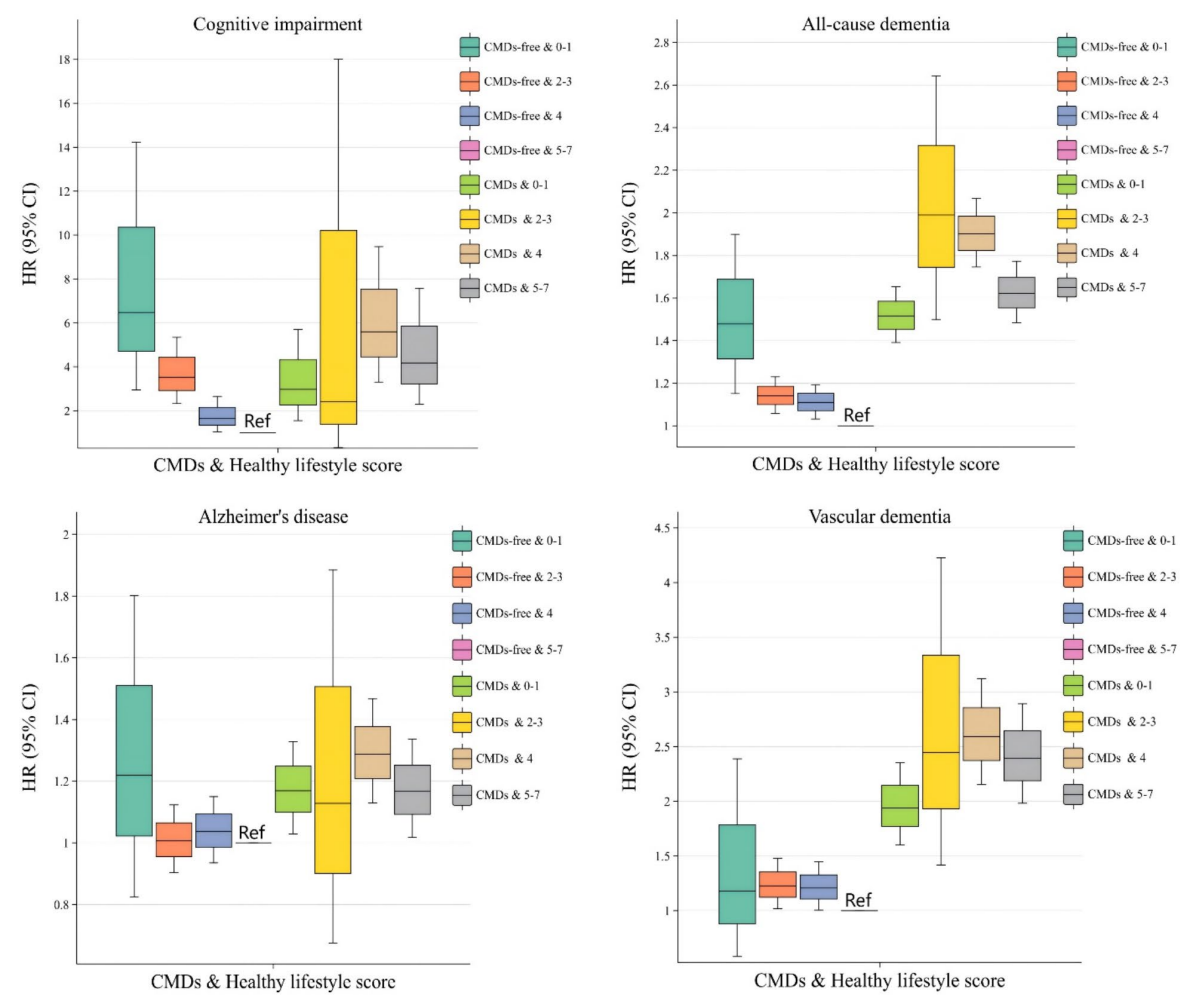
Association between healthy lifestyle score and risk of developing cognitive impairment and several dementia subtypes in patients with cardiometabolic disease. Abbreviations: CMDs, Cardiometabolic disease; HR, hazard ratios; CI, Confidence Intervals;In the model, we controlled for basic sociodemographic factors [age, sex, race, educational level, occupational status, TDI, BMI] and health-related concerns [APOE genotype; history of hypertension; history of depression; dyslipidemia; hypertriglyceridemia; aspirin use; lipid-lowering medication use; serum 25(OH)D levels].

When analyzed in combination with CMDs status and healthy lifestyle score factors, if patients with non-CMDs and those with CMDs maintained high healthy lifestyle scores, their risk of all-cause dementia, Alzheimer’s disease, and vascular dementia is lower (Fig. 2). The risk of mild cognitive impairment and different dementia types decreased as healthy lifestyle scores increased in individuals with or without CMDs. Those with scores of 5 to 7 and no CMDs had the lowest risk for mild cognitive impairment, all-cause dementia, and vascular dementia. The risk of developing mild cognitive impairment, all-cause dementia, Alzheimer’s disease, and vascular dementia was 2.973-fold, 1.516-fold, 1.169-fold, and 1.940-fold higher, respectively, among those with CMDs and a healthy lifestyle score of 0 to 1 than among those without CMDs and a healthy lifestyle score of 5 to 7 (Table 6). The incidence rates per 1000 person-years for mild cognitive impairment, all-cause dementia, Alzheimer’s disease, and vascular dementia in patients with CMDs in the Healthy Lifestyle Score 0–1 subgroup were 0.095 (95% CI: 0.02, 0.39), 2.951 (95% CI: 2.28, 3.82), 0.912 (95% CI: 0.57, 1.45), and 0.814 (95% CI: 0.49, 1.33), respectively (Table S10).

**Table 6:** Correlation between CMDs status and healthy lifestyle combined variables and cognitive impairment and different subtypes of dementia.

CMDs status	Healthy lifestyle score	Adjust HR (95%CI)			
		Mild cognitive impairment	All-cause dementia	Alzheimer’s disease	Vascular dementia
CMDs-free	5–7	1(Ref)	1(Ref)	1(Ref)	1(Ref)
	0–1	6.478(2.949,14.233)	1.478(1.151,1.899)	1.219(0.825,1.802)	1.180(0.583,2.388)
	2–3	3.523(2.324,5.340)	1.141(1.058,1.230)	1.007(0.903,1.123)	1.227(1.018,1.479)
	4	1.655(1.032,2.655)	1.110(1.032,1.193)	1.037(0.935,1.150)	1.207(1.006,1.449)
CMDs	0–1	2.973(1.550,5.703)	1.516(1.391,1.653)	1.169(1.029,1.328)	1.940(1.600,2.352)
	2–3	2.422(0.326,18.010)	1.990(1.498,2.643)	1.128(0.675,1.885)	2.447(1.417,4.225)
	4	5.596(3.307,9.469)	1.901(1.746,2.068)	1.287(1.129,1.468)	2.592(2.153,3.120)
	5–7	4.162(2.290,7.565)	1.622(1.484,1.773)	1.167(1.018,1.336)	2.394(1.982,2.893)
	P value	< 0.001	< 0.001	0.005	< 0.001

Abbreviations: CMDs, Cardiometabolic disease; HR, hazard ratios; CI, Confidence Intervals.

In the model, we controlled for basic sociodemographic factors [age, sex, race, educational level, occupational status, TDI, BMI] and health-related concerns [APOE genotype; history of hypertension; history of depression; dyslipidemia; hypertriglyceridemia; aspirin use; lipid-lowering medication use; serum 25(OH)D levels].

In studies using CMDs presence or absence as a stratifying factor, a significant statistical association was discovered between a healthy lifestyle and the risk of incident all-cause dementia and vascular dementia in individuals with CMDs. The risk of developing all-cause dementia and vascular dementia was 1.314 and 1.354 times higher, respectively, in patients with CMDs scoring 0 to 1 on the Healthy Lifestyle Score than that in those with CMDs scoring 5 to 7 (Table 7). In addition, except for the healthy diet model, there were significant statistical associations between the remaining six healthy lifestyles and the risk of developing mild cognitive impairment or different subtypes of dementia. In terms of the magnitude of the effect of a healthy lifestyle on the risk of developing mild cognitive impairment and dementia, adopting a healthy lifestyle may have the greatest impact on reducing the risk of mild cognitive impairment (Table S11).

### Effect modification of ambient air pollution on mild cognitive impairment and dementia risk by lifestyle score in individuals with cardiometabolic diseases

**Table 7:** The influence of healthy lifestyle score on cognitive impairment and dementia risk of CMDs population and non-CMDs population stratified by CMDs status.

CMDs status	Healthy lifestyle score	Adjust HR (95%CI)			
		Mild cognitive impairment	All-cause dementia	Alzheimer’s disease	Vascular dementia
CMDs-free	5–7	1(Ref)	1(Ref)	1(Ref)	1(Ref)
	0–1	6.152(2.786,13.587)	1.504(1.170,1.932)	-	-
	2–3	3.421(2.251,5.199)	1.150(1.066,1.240)	-	-
	4	1.639(1.021,2.629)	1.113(1.034,1.197)	-	-
	P value	< 0.001	0.001	0.691	0.091
CMDs	5–7	1(Ref)	1(Ref)	1(Ref)	1(Ref)
	0–1	-	1.314(0.985,1.753)	-	1.354(0.787,2.330)
	2–3	-	1.253(1.139,1.379)	-	1.391(1.165,1.662)
	4	-	1.072(0.971,1.184)	-	1.256(1.046,1.509)
	P value	0.108	< 0.001	0.566	0.003

Abbreviations: CMDs, Cardiometabolic disease; HR, hazard ratios; CI, Confidence Intervals.

In the model, we controlled for basic sociodemographic factors [age, sex, race, educational level, occupational status, TDI, BMI] and health-related concerns [APOE genotype; history of hypertension; history of depression; dyslipidemia; hypertriglyceridemia; aspirin use; lipid-lowering medication use; serum 25(OH)D levels].

In CMDs patients with high levels of exposure to ambient air pollution, the risk of all-cause dementia may also decrease as the healthy lifestyle subgroup score increases [0 to 1 score subgroup: *HR = 3.049*,* 95% CI: 1.559*,* 5.965*; 2 to 3 score subgroup: *HR = 1.805*,* 95% CI: 1.534*,* 2.124*; 4 score subgroup: *HR = 1.525*,* 95% CI: 1.286*,* 1.808*; 5 to 7 score subgroup: *HR = 1.623*,* 95% CI: 1.383*,* 1.906*] (Table 8). Similar results were noted for the environmental contaminants PM_2.5_, and NO_X_ (Table S12). CMDs patients exposed to moderate air pollution have the highest risk of vascular dementia in the subgroups with healthy lifestyle scores of 2–3, 4, and 5–7. Ambient air pollutants had a more statistically significant adverse effect on the risk of vascular dementia in patients with CMDs than in the non-CMDs population. In addition, in the healthy lifestyle score 0 to 1 subgroup, no associations were found between patients with CMDs exposed to high levels of ambient air pollution and the risk of developing mild cognitive impairment, Alzheimer’s disease, and vascular dementia (Table 8).

**Table 8:** Effect modification of CMDs status and ambient air pollution combined variables on cognitive impairment and dementia risk by healthy lifestyle in individuals.

Lifestyle	CMDs status & Ambient air pollution	Adjust HR (95%CI)			
		Mild cognitive impairment	All-cause dementia	Alzheimer’s disease	Vascular dementia
0–1	CMDs-free + Low	1(Ref)	1(Ref)	1(Ref)	1(Ref)
	CMDs-free + Medium	-	1.521(0.765,3.023)	-	-
	CMDs-free + High	-	1.672(0.867,3.227)	-	-
	CMDs + Low	-	0.936(0.332,2.638)	-	-
	CMDs + Medium	-	2.191(1.040,4.616)	-	-
	CMDs + High	-	3.049(1.559,5.965)	-	-
	P value	0.719	0.010	0.416	0.417
2–3	CMDs-free + Low	1(Ref)	1(Ref)	1(Ref)	1(Ref)
	CMDs-free + Medium	-	1.083(0.937,1.252)	0.936(0.752,1.165)	1.308(0.937,1.828)
	CMDs-free + High	-	1.144(0.984,1.330)	1.263(1.023,1.560)	0.901(0.621,1.307)
	CMDs + Low	-	1.645(1.382,1.958)	1.179(0.894,1.554)	2.279(1.589,3.268)
	CMDs + Medium	-	1.986(1.701,2.320)	1.476(1.161,1.876)	2.717(1.952,3.782)
	CMDs + High	-	1.805(1.534,2.124)	1.432(1.123,1.825)	2.270(1.614,3.194)
	P value	0.505	< 0.001	< 0.001	< 0.001
4	CMDs-free + Low	1(Ref)	1(Ref)	1(Ref)	1(Ref)
	CMDs-free + Medium	2.170(0.900,5.235)	1.098(0.963,1.252)	-	0.978(0.713,1.341)
	CMDs-free + High	2.003(0.815,4.922)	1.011(0.873,1.171)	-	0.975(0.695,1.367)
	CMDs + Low	4.132(1.289,13.243)	1.438(1.213,1.705)	-	1.788(1.270,2.518)
	CMDs + Medium	2.694(0.775,9.359)	1.673(1.435,1.952)	-	2.171(1.587,2.969)
	CMDs + High	7.110(2.649,19.083)	1.525(1.286,1.808)	-	1.789(1.275,2.510)
	P value	0.003	< 0.001	0.236	< 0.001
5–7	CMDs-free + Low	1(Ref)	1(Ref)	1(Ref)	1(Ref)
	CMDs-free + Medium	-	1.091(0.972,1.226)	-	1.324(0.983,1.784)
	CMDs-free + High	-	1.150(1.010,1.309)	-	1.326(0.959,1.835)
	CMDs + Low	-	1.565(1.343,1.824)	-	1.945(1.355,2.793)
	CMDs + Medium	-	1.538(1.329,1.779)	-	2.872(2.089,3.950)
	CMDs + High	-	1.623(1.383,1.906)	-	2.315(1.620,3.308)
	P value	0.161	< 0.001	0.179	< 0.001

Abbreviations: CMDs, Cardiometabolic disease; HR, hazard ratios; CI, Confidence Intervals.

In the model, we controlled for basic sociodemographic factors [age, sex, race, educational level, occupational status, TDI, BMI] and health-related concerns [APOE genotype; history of hypertension; history of depression; dyslipidemia; hypertriglyceridemia; aspirin use; lipid-lowering medication use; serum 25(OH)D levels].

When analyzing the modulatory effect of a healthy lifestyle on the impact of ambient air pollution on mild cognitive impairment and different subtypes of dementia risk in the population without CMDs and CMDs, it was found that exposure to ambient air pollution may have had a greater effect on the risk of Alzheimer’s disease in non-CMDs patients in the 5 to 7 point subgroup compared to the 2 to 3 point subgroup of healthy lifestyle scores [2 to 3 score subgroup: *HR = 1.267*,* 95% CI: 1.025*,* 1.566*; 5 to 7 score subgroup: *HR = 1.328*,* 95% CI: 1.123*,* 1.572*] (Table 9). In addition, in the healthy lifestyle score 0 to 1 subgroup, a significant statistical association was found between PM_2.5_ and NO_X_ exposure and the risk of all-cause dementia in CMDs patients. In the healthy lifestyle score 2 to 3 subgroup, a significant statistical association was found between PM10 exposure and the risk of all-cause dementia and Alzheimer’s disease in CMDs patients (Table S13).

**Table 9:** Effect modification of ambient air pollution on cognitive impairment and dementia risk by healthy lifestyle in individuals when CMDs status is used as a stratification factor.

CMDs status	Lifestyle	Ambient air pollution	Adjust HR (95%CI)			
			Mild cognitive impairment	All-cause dementia	Alzheimer’s disease	Vascular dementia
CMDs-free	0–1	Low	1(Ref)	1(Ref)	1(Ref)	1(Ref)
		Medium	-	-	-	-
		High	-	-	-	-
		P value	0.886	0.334	0.634	0.818
	2–3	Low	1(Ref)	1(Ref)	1(Ref)	1(Ref)
		Medium	-	-	0.936(0.752,1.165)	-
		High	-	-	1.267(1.025,1.566)	-
		P value	0.191	0.159	0.008	0.064
	4	Low	1(Ref)	1(Ref)	1(Ref)	1(Ref)
		Medium	-	-	-	-
		High	-	-	-	-
		P value	0.228	0.288	0.345	0.999
	5–7	Low	1(Ref)	1(Ref)	1(Ref)	1(Ref)
		Medium	-	1.115(0.993,1.251)	1.084(0.922,1.274)	-
		High	-	1.235(1.091,1.397)	1.328(1.123,1.572)	-
		P value	0.532	0.004	0.003	0.393
CMDs	0–1	Low	1(Ref)	1(Ref)	1(Ref)	1(Ref)
		Medium	-	2.256(0.825,6.166)	-	-
		High	-	3.484(1.345,9.027)	-	-
		P value	0.159	0.027	0.130	0.075
	2–3	Low	1(Ref)	1(Ref)	1(Ref)	1(Ref)
		Medium	-	-	-	-
		High	-	-	-	-
		P value	0.573	0.079	0.390	0.288
	4	Low	1(Ref)	1(Ref)	1(Ref)	1(Ref)
		Medium	-	-	-	-
		High	-	-	-	-
		P value	0.474	0.131	0.157	0.317
	5–7	Low	1(Ref)	1(Ref)	1(Ref)	1(Ref)
		Medium	-	-	-	1.522(1.097,2.110)
		High	-	-	-	1.224(0.849,1.763)
		P value	0.100	0.994	0.277	0.038

Abbreviations: CMDs, Cardiometabolic disease; HR, hazard ratios; CI, Confidence Intervals.

In the model, we controlled for basic sociodemographic factors [age, sex, race, educational level, occupational status, TDI, BMI] and health-related concerns [APOE genotype; history of hypertension; history of depression; dyslipidemia; hypertriglyceridemia; aspirin use; lipid-lowering medication use; serum 25(OH)D levels].

At the same time, an interaction was found in this study between the scores for a healthy lifestyle and the exposure to ambient air pollution. Using low-level exposure to ambient air pollution and a healthy lifestyle score of 5 to 7 as the control group, we found that exposure to medium-level ambient air pollution and a healthy lifestyle score of 0 to 1 in non-CMDs population may have the greatest risk of mild cognitive impairment and all-cause dementia. In contrast, exposure to high levels of ambient air pollution and a healthy lifestyle score of 0 to 1 in the CMDs population may have the greatest risk of all-cause dementia and vascular dementia. These included a possible reduction in the risk of all-cause dementia and vascular dementia in patients with CMDs with increasing healthy lifestyle scores at constant levels of exposure to ambient air pollution (Table 10). Sensitivity analyses yielded similar results, further ensuring the reliability of the study (Table S14 to S25).

**Table 10:** Effects of ambient air pollution and healthy lifestyle score interactions on the risk of developing cognitive impairment and different dementia subtypes in patients with CMDs.

CMDs status	Ambient air pollution & Lifestyle	Adjust HR (95%CI)			
		Mild cognitive impairment	All-cause dementia	Alzheimer’s disease	Vascular dementia
CMDs-free	Low + 5–7	1(Ref)	1(Ref)	1(Ref)	1(Ref)
	medium + 0–1	4.665(1.132,19.228)	1.648(1.083,2.509)	-	-
	High + 0–1	4.355(1.539,12.325)	1.585(1.096,2.291)	-	-
	medium + 2–3	3.303(2.139,5.099)	1.131(1.020,1.253)	-	-
	High + 2–3	2.669(1.709,4.169)	1.198(1.077,1.333)	-	-
	medium + 4	1.681(0.974,2.901)	1.153(1.047,1.269)	-	-
	High + 4	1.277(0.711,2.292)	1.055(0.944,1.180)	-	-
	P value	< 0.001	< 0.001	0.147	0.098
CMDs	Low + 5–7	1(Ref)	1(Ref)	1(Ref)	1(Ref)
	medium + 0–1	-	1.305(0.796,2.139)	-	1.266(0.521,3.072)
	High + 0–1	-	1.598(1.101,2.320)	-	1.935(0.996,3.760)
	medium + 2–3	-	1.340(1.193,1.507)	-	1.450(1.175,1.789)
	High + 2–3	-	1.205(1.066,1.362)	-	1.243(0.995,1.554)
	medium + 4	-	1.139(1.006,1.291)	-	1.324(1.063,1.650)
	High + 4	-	1.005(0.874,1.156)	-	1.108(0.862,1.425)
	P value	0.230	< 0.001	0.387	0.006

Abbreviations: CMDs, Cardiometabolic disease; HR, hazard ratios; CI, Confidence Intervals.

In the model, we controlled for basic sociodemographic factors [age, sex, race, educational level, occupational status, TDI, BMI] and health-related concerns [APOE genotype; history of hypertension; history of depression; dyslipidemia; hypertriglyceridemia; aspirin use; lipid-lowering medication use; serum 25(OH)D levels].

## Discussion

This study found that patients with CMDs may have a higher risk of developing mild cognitive impairment and dementia than those without CMDs. The risk of developing mild cognitive impairment and dementia in the population seems to increase with the increase in the number of CMDs. There appears to be a statistically significant association between high levels of ambient air pollution, unhealthy lifestyles, and a higher risk of developing mild cognitive impairment and dementia in the CMDs population. For example, people with CMDs are 1.086 times more likely to develop vascular dementia if they are exposed to the highest quartile of ambient air pollution than if they are exposed to the lowest quartile. The risk of developing mild cognitive impairment and different dementia types may be decreased as healthy lifestyle scores increase in individuals with or without CMDs. People with CMDs were 1.314 and 1.354 times more likely to develop all-cause dementia and vascular dementia, respectively, if they had a healthy lifestyle score of 5 to 7 than if they had a score of 0 to 1. A healthy lifestyle may have an effect modifier role in the association between ambient air pollution and the risk of mild cognitive impairment and the development of various dementia subtypes in patients with CMDs. As the healthy lifestyle score rose, the risk of developing all-cause dementia may be reduced in CMDs patients with high levels of ambient air pollution exposure. Therefore, maybe people with CMDs can lessen the impact of ambient air pollution on their risk of developing mild cognitive impairment and dementia by improving their lifestyle. Sensitivity analyses yielded similar results, further ensuring the reliability of the study.

This study found that the risk of developing mild cognitive impairment and dementia in the population seems to increase with the increase in the number of CMDs. Among people with three kinds of CMDs, all-cause dementia, Alzheimer’s disease, and vascular dementia seem to have the highest risk of developing. The results suggest that there may be an additive effect in the relationship between CMDs and the risk of developing cognitive impairment and dementia, which is consistent with previous studies^29^. Furthermore, having two or more CMDs will interact with each other, which may increase the risk of adverse health consequences^10^. Multimorbidity of CMDs accelerates cognitive decline and increases the risk of conversion to dementia in people without dementia^9^. Therefore, our study highlights the importance of delaying or halting the development of cardiovascular disease in people with diabetes. This may lead to greater health benefits in preventing the development of diabetic complications such as dementia, and vice versa.

As far as we know, previous studies on the influence of air pollution on cognitive dysfunction and the risk of dementia mainly focused on normal people^30^. There is a lack of corresponding research on the susceptible population CMDs population. Our research addresses this gap. Our study found that high exposure to ambient air pollution may increase the risk of developing mild cognitive impairment and dementia in patients with CMDs. This may be because ambient air pollution leads to inflammatory responses and oxidative stress, which trigger the onset and development of disease^24^. Ambient air pollutants can travel through the bloodstream to the brain, causing systemic inflammation^23^. During this process, the blood-brain barrier is compromised, microglial cells are activated^23^, the body releases a variety of pro-inflammatory mediators, and neural-immune interactions are triggered, leading to oxidative stress and further exacerbating the inflammatory response^24^. This can therefore lead to metabolic dysfunction diseases such as hyperlipidemia and atherosclerosis^31^, increasing the risk of cardiovascular metabolic diseases such as stroke^23^, which in turn increases the population’s risk of developing neurodegenerative diseases such as dementia^25,26^. Thus, neuroinflammation and oxidative stress may be key factors in the relationship between ambient air pollution and CMDs and dementia.

This study found that the risk of developing mild cognitive impairment and dementia may be decreased as healthy lifestyle scores increase in people with or without CMDs. Previous studies have shown that adopting a healthy lifestyle can delay the onset of dementia in people with CMDs by up to 3.50 years^32^. So, adherence to a healthy lifestyle may counteract the increased risk of dementia due to cardiometabolic multimorbidity, which is consistent with previous studies^33^. This may be for the following reasons. First, unhealthy lifestyles, including sedentary lifestyles, excessive alcohol consumption, and smoking, lead to vascular damage and inflammatory responses in the body, which accelerate the development of cognitive impairment and dementia in the context of cardiometabolic disease^34^. In contrast, adherence to a healthy lifestyle reduces the risk of cardiovascular disease^35^ and dementia^33^ through mechanisms such as reducing inflammatory responses, inhibiting oxidative stress, increasing cerebral blood flow, and reducing amyloid aggregation and neuroinflammatory plaques^27^. Secondly, a healthy lifestyle can postpone the onset of dementia by replenishing levels of neurotrophic factors and cytokines levels through regulating the mechanisms that trigger neuronal damage and neurodegenerative pathologies^36^. Thirdly, adopting a healthy lifestyle may reduce the risk of cognitive decline and delaying the onset of dementia^37^ by increasing the efficiency of neural networks, thereby increasing cognitive reserve, further increasing resistance to the effects of neuropathology, and increasing resilience to the effects of neuropathology^38^. Moreover, adopting a healthy lifestyle assists individuals with CMDs in modifying their health attitudes, engaging actively in their treatment, managing blood glucose and blood pressure, monitoring for complications, minimizing exposure to cardiovascular and cerebrovascular risk factors, and decreasing the risk of cognitive impairment and dementia^39^.Studies have shown that healthy diets reduce dementia risk by reducing inflammation and oxidative stress^40^. However, this study did not find a significant statistical association between healthy dietary patterns and the risk of developing mild cognitive impairment and dementia in people with CMDs. This could be for the following reasons. Firstly, a previous UK Biobank study indicated a negative link between cognitive function and fruit/vegetable intake and a positive one with meat consumption^41^. In contrast, our analysis focused on high vegetable/fruit and low meat consumption patterns, potentially affecting the relationship between cognitive function decline and dementia. Next, a U-shaped relationship exists between food intake and the risk of developing dementia^42^. This study evaluates dietary patterns by looking at the highest or lowest intake of different foods and classifies some high-risk groups for dementia as having healthy dietary patterns. Thirdly, other important dietary factors that are strongly associated with the risk of developing dementia, such as nuts^43^, legumes^44^, and olive oil^45^, were not included in the assessment of healthy eating patterns, which affected the assessment of the association between a healthy diet and the risk of developing dementia.

This study found that a healthy lifestyle may reduce the effects of ambient air pollution on the risk of developing dementia in people with CMDs. This is because ambient air pollution triggers an inflammatory response and oxidative stress in the body, which contribute to the development of disease^24^. Maintaining a healthy lifestyle can help reduce inflammation, inhibit oxidative stress, increase cerebral blood flow, and reduce amyloid aggregation^27^, thereby reducing the risk of cardiovascular disease^35^ and dementia^33^. For example, adequate sleep not only boosts antioxidant mechanisms by eliminating reactive oxygen species from the body but also helps regulate the inflammatory response to ambient air pollution exposure by enhancing immune defenses^46^. In addition, the adverse effects of ambient air pollution on cognitive function can be offset by a healthy diet rich in foods and nutrients that promote antioxidant and anti-inflammatory activity^47^. At an individual level, it may be difficult to control the level of ambient air pollution, but lifestyle changes may reduce its adverse effects on health. Therefore, the earlier a healthy lifestyle is adhered to in order to prevent or delay the onset of dementia, the greater health benefits are likely to be.

To our knowledge, few studies have simultaneously investigated the association of ambient air pollution and lifestyle with the risk of mild cognitive impairment and dementia in patients with CMDs. The novelties of this study may include the following. Firstly, this is the first study to use data from a large prospective cohort study to examine the effects of ambient air pollution on the risk of mild cognitive impairment and dementia development in patients with CMDs. Secondly, the present study may further demonstrate the additive effect of different cardiometabolic diseases in causing the risk of developing mild cognitive impairment and dementia. Thirdly, the research suggested that maintaining a healthy lifestyle may reduce the negative effects of ambient air pollution on the risk of developing dementia in people with CMDs. At an individual level, it may be difficult to control the level of ambient air pollution, but lifestyle changes may reduce its adverse effects on health. Therefore, maybe to prevent or delay the onset of dementia in old age, adherence to a healthy lifestyle early in life may have greater health benefits. In addition, the large sample size, long-term follow-up, and rigorously defined variables contribute to the credibility and accuracy of this research.

However, this study has limitations. Firstly, we did not take into account the variation in pollution levels over time, focusing instead on the average ambient air pollution concentration in 2010. However, previous research suggests that ambient air pollution levels remained fairly constant throughout the UK Biobank follow-up period^48^. Secondly, the baseline data, which rely on self-reported lifestyle factors, may have undergone unknown changes during follow-up, so there is a degree of information bias within the overall lifestyle data. Additionally, we might subjectively view smoking status as a lifestyle indicator without considering the individual’s health based on their daily cigarette consumption. Finally, even after adjusting for many potential confounders, residual confounding from unmeasured or unknown variables could still affect our analyses.

## Methods

### Study design and population

The UK Biobank is a comprehensive biomedical database containing detailed genetic and health information on around 500,000 participants (aged 39–74) recruited between April 2006 and December 2010 from 22 assessment centres in England, Wales and Scotland^49^. In addition, the UK Biobank follows the ethical principles of the Declaration of Helsinki and has been approved by the Northwest Multicenter Research Ethics Committee, allowing researchers to work within the approved scope without further ethical review.

Baseline data from 502,370 participants in the UK Biobank, collected between 2006 and 2010, were used primarily for this study. Due to a lack of baseline data, four individuals were excluded from the initial analysis. Second, 5357 participants with type 1 diabetes at baseline, 201 participants with mild cognitive impairment at baseline, and 226 participants with dementia at baseline were excluded. In addition, we also excluded 40,852 participants without information on ambient air pollution and 17,049 participants without information on lifestyle. There were 438,681 subjects in the final cohort, and these subjects were followed for an average of 15.12 years until the study ended on 1 April 2024 (See Figure S1).

### Assessment of cardiometabolic diseases

CMDs encompass T2DM, stroke, and CHD. We used UK Biobank medical history, hospital records, and self-reported information to identify participants with baseline T2DM, stroke, and CHD. Refer to Table S1 for details.

### Assessment of ambient air pollution and lifestyle factors

The UK Biobank database estimates annual mean values of PM_2.5_, PM_10_, PM_2.5−10_, NO_2_, and NO_X_ using land use regression (LUR) modeling^50,51^. In this study, we used the ambient air pollution data collected in 2010 to evaluate the exposure level of individual baseline ambient air pollution. Atmospheric pollutants were categorized into consecutive interquartile ranges (IQRs) and quartiles based on their distributions. Air pollutants were then analyzed using Latent Category Analysis (LCA) to create a latent variable representing the overall level of exposure to ambient air pollution. The new latent variable represents high, moderate, and low levels of ambient air pollution exposure (See Table S2 to 3 and Figure S2 for details.).

Healthy lifestyle scores were assessed using seven lifestyle items from a structured baseline questionnaire: diet, smoking, alcohol, physical activity, social relationships, sedentary behavior, and sleep patterns. In addition, according to the Pearson correlation coefficient test found no significant collinearity among the seven lifestyles. Meeting the optimal intake of at least four of the seven food groups is considered a healthy eating pattern, according to the US Dietary Guidelines for Cardiovascular Health^41^. Never smoking is seen as a healthy lifestyle. Moderate drinking, defined as 0–28 g/day for men and 0–14 g/day for women^52^, effectively reduces the risk of dementia^18^. Healthy physical activity is measured by regular moderate and vigorous physical activity each week^53^. Healthy social relationships were measured according to the Social Connectedness Index Score, in which active and moderately active participants were considered to have healthy social relationships^54^. Sedentary behavior was measured based on activities such as driving, using a computer, and watching TV, with less than four hours being considered healthy sedentary behavior^55^. Sleep patterns were assessed according to five principles, including appropriate sleep duration and sleep habits, and healthy sleep patterns were defined as meeting at least four of these principles^56^. Give each lifestyle 1 point if it is considered healthy and 0 points if it is not. The Healthy Lifestyle Score ranges from 0 to 7, with higher scores indicating that individuals are living a healthier lifestyle. In order to avoid extremes in rare cases, the lifestyle scores are divided into four groups (0 to 1, 2 to 3, 4 and 5 to 7). See Table S4 for the definition of lifestyle.

### Assessment of outcome

Diagnoses of mild cognitive impairment and dementia were made using health-related outcomes defined by the UK Biobank preprocessing algorithm^57^, based on primary care, hospital admission, and death registration data during follow-up. Data on health outcomes were published in 2018, with data dynamically updated until the end of this study in April 2024. Subsequently, the International Classification of Diseases (ICD) 9th and 10th editions were utilized for categorizing mild cognitive impairment and dementia. The dependent variables included mild cognitive impairment and dementia, with dementia encompassing all-cause dementia, Alzheimer’s disease, and vascular dementia. Patients with all-cause dementia are those with all types of dementia, including Alzheimer’s disease, vascular dementia, frontotemporal dementia and some undetermined types of dementia. Using routinely collected medical records, the UK Biobank showed high positive predictive value, sensitivity, and specificity for detecting dementia events^58^. For more information on coding mild cognitive impairment and dementia, refer to Table S5 in the supplementary materials.

### Assessment of covariates

Confounders of potential socio-demographic, economic, and health problems were identified through questionnaires in the baseline survey. Confounders included age, sex (male and female), race (white, Asian, black, and other), education level (college or university degree, A level/AS levels or equivalent, O level/GCSEs or equivalent, Professional Qualifications, and None of the above), occupation (employed, unemployed and retired), Townsend Deprivation Index (TDI) (low, medium and high), body mass index (BMI) (< 18. 5, 18.5–24.9, 25-29.9 and ≥ 30 kg/m2), APOE genotype, history of hypertension, history of depression, dyslipidemia, hypertriglyceridemia, aspirin use status, lipid-lowering drug use status and serum 25(OH)D status (severely deficient, moderately deficient and deficient and above). Socioeconomic status was evaluated with the TDI, where a higher TDI indicates increased poverty levels^59^. Genotyping utilized the APOE single nucleotide polymorphisms rs7412 and rs429358 to detect carriers of the APOE ε4 allele^60^. Medical records, self-reports, biological samples, and death records were used to extract other covariate information. Please refer to Table S6 in the annex for the sources of some covariant components and their evaluation criteria.

### Statistical analyses

Characteristics of patients with CMDs were expressed as means and standard deviations (SD) for continuous variables and percentages for categorical variables. LCA was employed to examine patterns of multiple ambient air pollutants, with six latent profile models performed. Several criteria were used to test the goodness of fit for the selection of latent classes, including Aikaike’s Information Criterion (AIC), Bayesian Information Criterion (BIC), Adjusted Bayesian Information Criterion (aBIC), Entropy, Lo-Mendell-Rubin Likelihood Ratio Test (LMRT) and Bootstrapping Likelihood Ratio Test^61^. Models with smaller AICs, BICs and aBICs were regarded as having a better fit^62^. Person-years at risk were calculated for each participant from the date of recruitment until the occurrence of dementia, death, loss to follow-up, or the cut-off date (1 April 2024), whichever came first. The proportional risk assumption was validated by Schoenfeld residuals. This study aimed to investigate the association between exposure to ambient air pollution and adherence to a healthy lifestyle and the risk of mild cognitive impairment and dementia in patients with CMDs. Firstly, to investigate the relationship between ambient air pollution exposure and the risk of mild cognitive impairment and dementia in CMDs patients. Secondly, to examine the link between adherence to a healthy lifestyle and the risk of mild cognitive impairment and dementia in CMDs patients. Thirdly, the effect of adherence to a healthy lifestyle on the association between exposure to ambient air pollution and the risk of mild cognitive impairment and dementia in patients with CMDs was examined. In addition, we also studied the correlation between the interaction between ambient air pollution exposure and a healthy lifestyle and the risk of mild cognitive impairment and dementia in CMDs patients. The entire analysis was primarily performed using the Cox proportional risk regression model, and the corresponding hazard ratios (HRs) and 95% confidence intervals (CIs) were calculated. In the model, we controlled for basic sociodemographic factors [age, sex, race, educational level, occupational status, TDI, BMI] and health-related concerns [APOE genotype; history of hypertension; history of depression; dyslipidemia; hypertriglyceridemia; aspirin use; lipid-lowering medication use; serum 25(OH)D levels].

To assess the robustness of our results, we have carried out three sensitivity analyses. First, to minimize reverse causality, we excluded participants who had developed dementia within two years. Second, insulin resistance is an independent risk factor for cardiometabolic disease and the development of dementia, and we further adjusted for indicator variables representing insulin resistance. Third, to account for the effect of missing data on the results, we excluded missing parts of confounding variables.

All statistical analyses were performed using Stata software version 17 and R version 4.0.2, and statistical significance (two-sided) was defined as a P value < 0.05.

## Conclusions

The risk of developing mild cognitive impairment and dementia in the population seems to increase with the increase in the number of CMDs. In the CMDs population, high levels of ambient air pollution exposure and unhealthy lifestyles seem to increase the risk of mild cognitive impairment and dementia. Adopting a healthy lifestyle may have an effect modifier role in the association between ambient air pollution and the risk of mild cognitive impairment and the development of dementia in patients with CMDs. Therefore, it seems that we can reduce the negative impact of ambient air pollution on mild cognitive impairment and dementia risk of the CMDs population by making lifestyle healthier.

The datasets analyzed in this study are available from the corresponding author upon reasonable request.

## Electronic Supplementary Material

Below is the link to the electronic supplementary material.

Supplementary Material 1

## Data Availability

The data used in this paper comes from survey data from the UK Biobank database, which can be applied through the official website at https://www.ukbiobank.ac.uk/. The datasets analyzed in this study are available from the corresponding author upon reasonable request.
