# Supplementary material for: Association between air pollution and lifestyle with the risk of developing mild cognitive impairment and dementia in individuals with cardiometabolic diseases
Source: Sci Rep. 2025 Jan 15;15:2089. doi: 10.1038/s41598-024-83607-w (PMC11736067; doi:10.1038/s41598-024-83607-w)

**Association between air pollution and lifestyle with the risk of developing mild cognitive impairment and dementia in individuals with cardiometabolic diseases**

**Supplementary information**

**Table S1. Definition of Cardiometabolic disease in the UK Biobank**

**Table S2. Definition of Ambient air pollution in the UK Biobank**

**Table S3. Comparison of indexes in analysis of potential categories of ambient air pollutants**

**Table S4. Definition of Lifestyle in the UK Biobank**

**Table S5. Definition of mild cognitive impairment and several dementia subtypes in the UK Biobank**

**Table S6. Definition of covariates in the UK Biobank**

**Table S7. Correlation between CMDs status and ambient air pollutants combined variables and mild cognitive impairment and different subtypes of dementia.**

**Table S8. Ambient air pollution causes the incidence of mild cognitive impairment and different subtypes of dementia in patients with CMDs.**

**Table S9. There is an association between ambient air pollutants and mild cognitive impairment and different subtypes of dementia in patients with CMDs when CMDs are used as a stratification factor.**

**Table S10. Healthy lifestyle score causes the incidence of mild cognitive impairment and different subtypes of dementia in patients with CMDs.**

**Table S11. There is an association between healthy lifestyle and mild cognitive impairment and different subtypes of dementia in patients with CMDs when CMDs are used as a stratification factor.**

**Table S12. Effect modification of ambient air pollutants on mild cognitive impairment and different subtypes of dementia risk by lifestyle score in patients with CMDs when considering the combined factors of CMDs status and ambient air pollutants.**

**Table S13. Effect modification of ambient air pollutants on mild cognitive impairment and different subtypes of dementia risk by lifestyle score in patients with CMDs when CMDs are used as a stratification factor.**

**Table S14. After excluding patients with dementia in the first two years, the association between CMDs status and air pollution combined variables and mild cognitive impairment and different subtypes of dementia, and the association between air pollution and mild cognitive impairment and different subtypes of dementia in patients with CMDs when CMDs is used as a stratification factor.**

**Table S15. After excluding patients with dementia in the first two years, the association between CMDs status and healthy lifestyle combined variables and mild cognitive impairment and different subtypes of dementia, and the association between healthy lifestyle score and mild cognitive impairment and different subtypes of dementia in patients with CMDs when CMDs is used as a stratification factor.**

**Table S16. After excluding patients with dementia in the first two years, effect modification of CMD status and ambient air pollution combined variables on mild cognitive impairment and dementia risk by healthy lifestyle in individuals.**

**Table S17. After excluding patients with dementia in the first two years, effect modification of ambient air pollution on mild cognitive impairment and dementia risk by healthy lifestyle in individuals when CMDs status are used as a stratification factor.**

**Table S18. After further adjustment for insulin resistance index, the association between CMDs status and air pollution combined variables and mild cognitive impairment and different subtypes of dementia, and the association between air pollution and mild cognitive impairment and different subtypes of dementia in patients with CMDs when CMDs is used as a stratification factor.**

**Table S19. After further adjustment for insulin resistance index, the association between CMDs status and healthy lifestyle combined variables and mild cognitive impairment and different subtypes of dementia, and the association between healthy lifestyle score and mild cognitive impairment and different subtypes of dementia in patients with CMDs when CMDs is used as a stratification factor.**

**Table S20. After further adjustment for insulin resistance index, effect modification of CMD status and ambient air pollution combined variables on mild cognitive impairment and dementia risk by healthy lifestyle in individuals.**

**Table S21. After further adjustment for insulin resistance index, effect modification of ambient air pollution on mild cognitive impairment and dementia risk by healthy lifestyle in individuals when CMDs status are used as a stratification factor.**

**Table S22. After removing missing values of covariates, the association between CMDs status and air pollution combined variables and mild cognitive impairment and different subtypes of dementia, and the association between air pollution and mild cognitive impairment and different subtypes of dementia in patients with CMDs when CMDs is used as a stratification factor.**

**Table S23. After removing missing values of covariates, the association between CMDs status and healthy lifestyle combined variables and mild cognitive impairment and different subtypes of dementia, and the association between healthy lifestyle score and mild cognitive impairment and different subtypes of dementia in patients with CMDs when CMDs is used as a stratification factor.**

**Table S24. After removing missing values of covariates, effect modification of CMD status and ambient air pollution combined variables on mild cognitive impairment and dementia risk by healthy lifestyle in individuals.**

**Table S25. After removing missing values of covariates, effect modification of ambient air pollution on mild cognitive impairment and dementia risk by healthy lifestyle in individuals when CMDs status are used as a stratification factor.**

**Figure S1.** **Flowchart of participants included in the analysis**

**Figure S2.** **Category difference of comprehensive variables of ambient air pollution**

Table S1. Definition of Cardiometabolic disease in the UK Biobank

| Variables | UK Biobank field code | Definition | Note |
| --- | --- | --- | --- |
| T2DM | Diagnoses - ICD10: E11, E14; Date first reported: 130708, 130714; Self-reported field code:2443, 2976 6153, 6177, 2986,120007, 20002; Blood biochemistry examination: 30740, 30750; | According to the American Diabetes Association 2022 guidelines, the definition of diabetes includes a baseline HbA1c ≥6.5% (48 mmol/mol) or a physician diagnosis of diabetes or use of insulin and glucose-lowering medications or baseline Glucose ≥11.1 mmol/L. | The UK Biobank database combines self-reported and hospital registry data with HbA1c levels measured by high performance liquid chromatography (HPLC) using the Bio-Rad VARIANT II Turbo to provide baseline information on people with type 2 diabetes. |
| Stroke | Diagnoses - ICD10:I60, I61, I63; Date first reported: Stroke:131368, 42006; Ischaemic stroke: 42008; Cerebral infarction:131366; Subarachnoid hemorrhage: 131360, 42012; Cerebral hemorrhage: 131362,42010; | We classify stroke subtypes as ischemic stroke, cerebral hemorrhage or subarachnoid hemorrhage. | Stroke events in the British biological sample bank are based on medical history and links with hospitalization and mortality data. We used stroke variables provided by the British biological sample bank, which were created by combining information from these different data sources. |
| CHD | Diagnoses - ICD10:I20, I21, I22, I23, I24, I25; Date first reported: 131296, 131298, 131300, 131302, 131304, 131306; | We divide the subtypes of coronary heart disease into angina pectoris and acute myocardial infarction and Subsequent myocardial infarction and Certain current complications following acute myocardial infarction and Other acute ischaemic heart diseases and Chronic ischaemic heart disease. | Coronary heart disease events in the British biological sample bank are based on medical history and are related to hospitalization and mortality data. We used coronary heart disease variables provided by British biological sample bank, which were created by combining information from these different data sources. |

Abbreviations: T2DM, type 2 diabetes mellitus; CHD, coronary heart disease;

Table S2. Definition of Ambient air pollution in the UK Biobank

| Ambient air pollution factor | UK Biobank field code | Definition | Latent class analysis(LCA) | Note |
| --- | --- | --- | --- | --- |
| PM_2.5_ | 24006 | Atmospheric pollutants were categorized into consecutive interquartile ranges (IQRs) and quartiles based on their distributions. Following that, latent class analysis (LCA) was conducted to create a novel latent variable. The study outcomes revealed three potential levels illustrating high, moderate, and low ambient air pollution patterns. | LCA was employed to examine patterns of multiple ambient air pollutants, with six latent profile models performed. The appropriate number of subgroups was determined based on a range of criteria including AIC, BIC, aBIC, Entropy, LMRT, and BLRT. A model was considered a better fit with smaller AIC, BIC, and aBIC values. The larger the entropy value, the more accurate the class classification, with values ≥ 0.8 indicating a good class solution. | Estimated by the Small Area Health Statistics Unit as part of the BioSHaRE-EU Environmental Determinants of Health Project, and calculated using a Land Use Regression (LUR) model developed as part of the European Study of Cohorts for Air Pollution Effects (ESCAPE) and represented annual averages of air pollution in 2010 for the reported residence at enrollment |
| PM_2.5-10_ | 24008 |  |  |  |
| PM_10_ | 24005 |  |  |  |
| NO_2_ | 24003 |  |  |  |
| NO_X_ | 24004 |  |  |  |

Abbreviations: PM_2.5_, particulate matter with aerodynamic diameter ≤2.5 µm; PM_10_, particulate matter with an aerodynamic diameter ≤10 µm; PM_2.5–10_, particulate matter with an aerodynamic diameter between 2.5 and 10 µm; NO_2_, nitrogen dioxide; NO_X_, nitrogen oxides; AIC, Aikaike's Information Criterion; BIC, Bayesian Information Criterion; aBIC, adjusted Bayesian Information Criterion; LMRT, Lo-Mendell Rubin Likelihood Ratio Test; BLRT, Bootstrap Likelihood Ratio Test;

Table S3. Comparison of indexes in analysis of potential categories of ambient air pollutants

| Number of categories | AIC | BIC | aBIC | LMRT | BLRT | Entropy | Category probability |
| --- | --- | --- | --- | --- | --- | --- | --- |
|  |  |  |  |  |  |  |  |
| 1 | 6716040.774 | 6716150.690 | 6716118.909 |  |  |  | 1.000 |
| 2 | 5686306.741 | 5686482.605 | 5686431.756 | 0.000 | 0.000 | 0.903 | 49.18%/50.82% |
| 3 | 5327747.823 | 5327989.636 | 5327919.719 | 0.000 | 0.000 | 0.896 | 36.14%/31.09%/32.77% |
| 4 | 5178857.697 | 5179165.459 | 5179076.474 | 0.000 | 0.000 | 0.890 | 24.36%/16.66%/31.30%/27.68% |
| 5 | 5049074.121 | 5049447.833 | 5049339.780 | 0.000 | 0.000 | 0.870 | 23.61%/26.96%/11.12%/13.09%/25.22% |
| 6 | 4896966.276 | 4897405.937 | 4897278.815 | 0.000 | 0.000 | 0.888 | 6.67%/17.84%/22.74%/17.57%/12.64%/22.54% |

Abbreviations: AIC, Aikaike's Information Criterion; BIC, Bayesian Information Criterion; aBIC, adjusted Bayesian Information Criterion; LMRT, Lo-Mendell Rubin Likelihood Ratio Test; BLRT, Bootstrap Likelihood Ratio Test;

Table S4. Definition of Lifestyle in the UK Biobank

| Variables | UK Biobank field code | Definition | Note |
| --- | --- | --- | --- |
| smoking status | 20116 | never, former, and current | Self-report and hospital registration records |
| dietary patterns | 1289, 1299,1309, 1319, 1329, 1339,1349,1369,1379,1389,1438,1458 | 1. Fruits: ≥ 3 servings/day; 2. Vegetables: ≥ 3 servings/day; 3. Whole grains: ≥ 3 servings/day; 4. (Shell) Fish: ≥2 servings/week; 5. Refined grains ≤1.5 servings/day; 6. Processed meats ≤1 serving/week; 7. Unprocessed meats ≤1.5 serving/week  "0-1 poor dietary pattern" ,"2-3 medium dietary pattern", ">3 ideal dietary pattern" | In this study, the British Dietetic Survey food frequency questionnaire (FFQ) was used to calculate a healthy eating score, with higher values indicating higher intake. The UKB FFQ has data on the reporting of their daily food intake (https://biobank.ndph.ox.ac.uk/ukb/label.cgi?id=100052). We used the Healthy Diet score. The diet score includes intake of vegetables (cooked vegetables + salads/raw vegetables), fruit (fresh fruit + dried fruit), unprocessed red meat (beef + lamb + pork), fish (fatty fish + non-fatty fish), Whole grains(Cereal intake(Cereal type>=1)),and Refine grain(Bread intake(Bread type>=1)).Frequency categories of meat and fish were recoded: ‘never’= 0, ‘less than once a week’ =0.5, ‘once a week’ = 1, ‘2–4 times a week’=3, ‘5–6 times a week’=5.5 and ‘once or more daily’=7.Amount of per serving was defined as: 1. fresh fruit: 1 piece; 2. dried fruit: 5 pieces; 3. cooked/raw vegetables: 3 heaped tablespoons;4. bran/oat/muesli cereal: 1 bowl/day; 5. wholemeal/wholegrain bread: 1 slice/day |
| alcohol consumption | 20117，1558，1568, 1578, 1588, 1598, 1608, 4407, 4418, 4429,4440, 4451, 4462, 5364 | UK Biobank Touchscreen questionnaire at baseline;US Dietary guidelines for Americans 2015-2020 of up to 1 drink/day for women and up to 2 drinks/day for men.To calculate drink-equivalents as per guidelines, multiply the volume in ounces by the alcohol content in percent and divide by 0.6 ounces of alcohol per drink-equivalent; then convert to grams: 1 drink-equivalent described as containing 14g of pure alcohol.125ml wine=0.85 drink-equivalents,4% ABV pint beer = 1.28 drink-equivalents,25ml spirits=0.57 drink-equivalents,50ml fortified wine= 0.56 drink-equivalents. | We multiplied the average weekly or monthly alcohol consumption by the appropriate conversion factor and then divided by 7 or 30 to get the daily alcohol consumption. |
| physical activity | 884, 894, 904, 914 | Meet at least one of the 2 following:  (i) Frequency: vigorous activity once and moderate physical activity at least 5 days per week (OR equivalent combination);  (ii) Time: vigorous activity for at least 75 minutes OR moderate activity for 150 minutes per week (OR equivalent combination) | Self-report |
| social relationships | 6160, 709, 1031 | The social contact index used in the UK Biobank was constructed from three questions: question 1, including yourself, how many people are living together in your household (we assigned 1 point for living alone); question 2, how often do you visit friends or family or have them visit you (we assigned 1 point for answering less than once a month); question 3, which of the following (sports club or gym, pub or social club, religious group, adult education class, other group activity) do you engage in once a week or more often (we assigned 1point for answering none of the above). | Individual scores were summed to obtain an overall score ranging from 0 to 3 and categorized as active (score = 0), moderately active (score = 1), and isolated (scores ≥2), according to a previous UK Biobank study. Being active and moderately active was considered as having frequent social contact. |
| sedentary behaviour | 1070, 1080, 1090 | Sedentary behavior is defined as any waking behavior while in a sitting, reclining, or lying posture with low energy expenditure (1.5 metabolic equivalents of task [METs] or lower). | Total sedentary behavior time was quantified by summing the time spent on watching TV, using a computer (not at work), and driving. We categorized sedentary behavior time into 4 groups: less than 2 h/d, 2 to 3 h/d, 4 to 5 h/d, and 6 h/d or more |
| sleep patterns | 1160, 1180, 1200, 1210, 1220 | Meet at least 4 of the following 5 patterns: (i) early chronotype ('morning' or 'morning than evening'); (ii) sleep 7–8 hours per day;  (iii) reported never or rarely insomnia symptoms;  (iv) no self-reported snoring;  (v) no frequent daytime sleepiness ('never/rarely' or 'sometimes') | All component scores were summed to obtain a healthy sleep score ranging from 0 to 5, with higher scores indicating a healthier sleep pattern. We then define the overall sleep patterns as ‘healthy sleep pattern’ (healthy sleep score ≥4), ‘intermediate sleep pattern’ (2≤healthy sleep score ≤3), and ‘poor sleep pattern’ (healthy sleep score ≤1) based on the healthy sleep score.  Five sleep factors over the last four weeks were considered: 1. chronotype: self-reported as a morning or evening person; 2. duration: hours of sleep per 24 hours; 3. insomnia: having trouble falling asleep at night or waking up in the middle of the night; 4. snoring: ever complained by a partner or close relative or friend about snoring; 5. excessive daytime sleepiness: frequency of dozing off or falling asleep involuntarily during the daytime. |

Table S5. Definition of mild cognitive impairment and several dementia subtypes in the UK Biobank

| Variables | ICD-9 diagnosis* | ICD-10 diagnosis* | Date first reported | Self-report |
| --- | --- | --- | --- | --- |
| Mild cognitive impairment |  | F06.7 |  | 20002(1263) |
| All-cause dementia | 290.0, 290.1, 290.2, 290.3, 290.4, 290.9, 291.2, 294.1, 294.2, 294.8, 294.9, 331.0, 331.1, 331.2, 331.8, 331.9 | G30, G30.0, G30.1, G30.8, G30.9, G31.0, G31.1, G31.8, F00, F00.0, F00.1, F00.2, F00.9, F01, F01.0, F01.1, F01.2, F01.3, F01.8, F01.9, F02, F02.0, F02.1, F02.2, F02.3, F02.4, F02.8, F03, F05.1, F10.6, A81.0, I67.3 | 42018, 42020, 42022, 42024, 130836, 130838, 130840, 130842, 131036 | 20002(1263) |
| Alzheimer's disease | 331 | G30, G30.0, G30.1, G30.8, G30.9, F00, F00.0, F00.1, F00.2, F00.9 | 42020, 130836,131036 | 20002(1263) |
| Vascular dementia | 290.4 | F01, F01.0, F01.2, F01.3, F01.8, F01.9, I67.3 | 42022,130838, | 20002(1263) |

Abbreviations: ICD, International Classification of Disease; * Refer to data codes used in UK Biobank Field ICD-9:41271,41203; ICD-10:41270,41202;

Table S6. Definition of covariates in the UK Biobank

| Variables | UK Biobank field code | Definition | Note |
| --- | --- | --- | --- |
| Townsend Poverty Index (TDI) | 22189 | The Townsend Poverty Index (TDI) is a composite measure of poverty based on postcode residence, using data on unemployment, household crowding, car and house ownership, with higher TDIs indicating higher levels of poverty. | SES was assessed using the TDI and divided into three levels using national cut-off points (high ≤-2.08; medium - 2.08-1.40; low ≥1.40). |
| APOE genotype | rs7412, rs429358 | Therefore, individuals with one or both ε4 alleles were classified as APOE ε4 carriers, while the rest were classified as APOE ε4 non-carriers. | The APOE ε4 allele has been recognized as a genetic risk factor for late-onset Alzheimer's disease. |
| depression | 20,433,130,894 | Age at first episode of depression; Date F32 first reported (depressive episode) | Self-report and hospital registration records |
| hypertension | 4079, 4080 | Two measures of blood pressure were collected, at least 1 minute apart, using an Omron HEM 7015-T automated sphygmomanometer. In the current study, a mean of both measures was used to determine systolic and diastolic blood pressure in mmHg. Hypertensive patients were identified according to hypertension diagnostic criteria of systolic blood pressure ≥140 mm of mercury (mmHg) or diastolic blood pressure ≥90 mmHg. | Diastolic blood pressure, automated reading  Systolic blood pressure, automated reading |
|  | 131286 | diagnosis of primary hypertension earlier than entry into the cohort. | Date I10 first reported (essential (primary) hypertension) |
|  | 6177, 6153 | Participants who responded “High blood pressure” to the touchscreen question “Has a doctor ever told you that you have had any of the following conditions?” or reported having hypertension during the verbal interview were defined as having doctor-diagnosed hypertension. Participants who responded “Blood pressure medications” to the touchscreen question “Do you regularly take any of the following medications?” or reported an antihypertensive medication (beta blockers, angiotensin-converting enzyme [ACE] inhibitors, angiotensin II receptor blockers, calcium channel blockers, alpha blockers, or diuretics) during the verbal interview were defined as using antihypertensive medication. | Medication for cholesterol, blood pressure or diabetes  Medication for cholesterol, blood pressure, diabetes, or take exogenous hormones |
| Dyslipidemia | 30760 | Dyslipidemia was defined as low high-density lipoprotein cholesterol (< 1.0 mmol/L (male), < 1.3 mmol/L (female)) | Self-report and hospital registration records |
| Hypertriglyceridemia | 30870 | Hypertriglyceridemia was defined as a triglyceride level ≥1.7 mmol/L. | Self-report and hospital registration records |
| Vitamin D | 30890 | Given that few participants had serum 25(OH)D ≥ 75 nmol/L in the current study population, serum vitamin D status was categorized into 3 groups: severely deficient (<25 nmol/L), moderately deficient (25 to <50 nmol/L), and insufficient and above (≥50 nmol/L), according to the Endocrine Society Clinical Practice Guidelines | Self-report and hospital registration records |

Abbreviations: TDI, Townsend deprivation index; APOE, apolipoprotein E;

Table S7. Correlation between CMDs status and ambient air pollutants combined variables and mild cognitive impairment and different subtypes of dementia.

| CMDs status | Ambient air pollution | Adjust HR (95% CI) | | | |
| --- | --- | --- | --- | --- | --- |
|  |  | Mild cognitive impairment | All-cause dementia | Alzheimer's disease | Vascular dementia |
| CMDs-free | PM_2.5_ Q 1 | 1(Ref) | 1(Ref) | 1(Ref) | 1(Ref) |
|  | PM_2.5_ Q 2 | 1.463(0.886,2.416) | 1.024(0.940,1.115) | 0.934(0.824,1.058) | 1.016(0.821,1.258) |
|  | PM_2.5_ Q 3 | 1.284(0.772,2.136) | 1.128(1.035,1.228) | 1.115(0.986,1.260) | 1.200(0.974,1.479) |
|  | PM_2.5_ Q 4 | 1.577(0.964,2.579) | 1.119(1.022,1.224) | 1.146(1.008,1.303) | 0.991(0.789,1.244) |
| CMDs | PM_2.5_ Q 1 | 2.377(1.149,4.917) | 1.527(1.376,1.696) | 1.128(0.959,1.326) | 2.048(1.641,2.558) |
|  | PM_2.5_ Q 2 | 2.802(1.460,5.378) | 1.692(1.531,1.871) | 1.273(1.092,1.484) | 2.293(1.850,2.843) |
|  | PM_2.5_ Q 3 | 2.272(1.161,4.445) | 1.723(1.559,1.904) | 1.268(1.088,1.478) | 2.227(1.794,2.764) |
|  | PM_2.5_ Q 4 | 3.028(1.666,5.504) | 1.685(1.520,1.868) | 1.295(1.108,1.513) | 1.972(1.572,2.474) |
|  | P value | 0.005 | <0.001 | <0.001 | <0.001 |
| CMDs-free | PM_2.5-10_ Q 1 | 1(Ref) | 1(Ref) | 1(Ref) | 1(Ref) |
|  | PM_2.5-10_ Q 2 | 1.225(0.788,1.902) | 1.028(0.946,1.118) | 1.027(0.911,1.159) | 0.984(0.795,1.218) |
|  | PM_2.5-10_ Q 3 | 0.977(0.617,1.547) | 1.022(0.938,1.113) | 0.981(0.867,1.111) | 1.070(0.865,1.324) |
|  | PM_2.5-10_ Q 4 | 1.060(0.675,1.665) | 0.980(0.898,1.069) | 1.007(0.890,1.140) | 1.064(0.859,1.318) |
| CMDs | PM_2.5-10_ Q 1 | 1.834(0.951,3.537) | 1.551(1.403,1.714) | 1.143(0.978,1.335) | 2.040(1.642,2.535) |
|  | PM_2.5-10_ Q 2 | 2.394(1.332,4.302) | 1.641(1.487,1.811) | 1.317(1.135,1.527) | 2.388(1.936,2.947) |
|  | PM_2.5-10_ Q 3 | 1.613(0.845,3.077) | 1.534(1.386,1.697) | 1.162(0.994,1.357) | 1.882(1.507,2.350) |
|  | PM_2.5-10_ Q 4 | 2.451(1.393,4.312) | 1.538(1.389,1.702) | 1.140(0.975,1.333) | 2.044(1.642,2.544) |
|  | P value | 0.006 | <0.001 | 0.003 | <0.001 |
| CMDs-free | PM_10_ Q 1 | 1(Ref) | 1(Ref) | 1(Ref) | 1(Ref) |
|  | PM_10_ Q 2 | - | 1.028(0.945,1.119) | 0.962(0.852,1.088) | 1.055(0.857,1.299) |
|  | PM_10_ Q 3 | - | 1.059(0.972,1.153) | 1.084(0.960,1.224) | 0.904(0.724,1.128) |
|  | PM_10_ Q 4 | - | 0.982(0.900,1.072) | 0.974(0.859,1.105) | 1.078(0.872,1.334) |
| CMDs | PM_10_ Q 1 | - | 1.459(1.316,1.618) | 1.168(0.999,1.366) | 1.912(1.534,2.383) |
|  | PM_10_ Q 2 | - | 1.701(1.543,1.876) | 1.152(0.988,1.344) | 2.328(1.889,2.870) |
|  | PM_10_ Q 3 | - | 1.614(1.460,1.784) | 1.307(1.125,1.519) | 1.910(1.533,2.381) |
|  | PM_10_ Q 4 | - | 1.545(1.395,1.712) | 1.136(0.970,1.329) | 2.048(1.646,2.549) |
|  | P value | 0.074 | <0.001 | 0.001 | <0.001 |
| CMDs-free | NO_2_ Q 1 | 1(Ref) | 1(Ref) | 1(Ref) | 1(Ref) |
|  | NO_2_ Q 2 | 1.615(0.956,2.729) | 1.079(0.992,1.174) | 1.047(0.928,1.182) | 1.095(0.889,1.348) |
|  | NO_2_ Q 3 | 1.683(1.002,2.825) | 1.100(1.010,1.199) | 1.040(0.918,1.177) | 1.099(0.889,1.359) |
|  | NO_2_ Q 4 | 1.677(0.994,2.829) | 1.083(0.986,1.189) | 1.107(0.969,1.265) | 0.993(0.786,1.254) |
| CMDs | NO_2_ Q 1 | 2.737(1.304,5.742) | 1.527(1.374,1.696) | 1.190(1.014,1.396) | 1.864(1.485,2.341) |
|  | NO_2_ Q 2 | 2.730(1.363,5.467) | 1.713(1.552,1.892) | 1.286(1.105,1.496) | 2.264(1.830,2.801) |
|  | NO_2_ Q 3 | 2.814(1.446,5.479) | 1.717(1.554,1.897) | 1.280(1.099,1.490) | 2.368(1.913,2.930) |
|  | NO_2_ Q 4 | 3.485(1.873,6.483) | 1.660(1.492,1.847) | 1.205(1.023,1.419) | 1.996(1.582,2.519) |
|  | P value | 0.004 | <0.001 | 0.006 | <0.001 |
| CMDs-free | NO_X_ Q 1 | 1(Ref) | 1(Ref) | 1(Ref) | 1(Ref) |
|  | NO_X_ Q 2 | 1.271(0.766,2.109) | 1.103(1.014,1.201) | 1.057(0.936,1.195) | 0.982(0.794,1.215) |
|  | NO_X_ Q 3 | 1.147(0.687,1.915) | 1.090(0.999,1.189) | 1.066(0.941,1.208) | 1.119(0.906,1.381) |
|  | NO_X_ Q 4 | 1.682(1.036,2.730) | 1.110(1.012,1.216) | 1.182(1.037,1.346) | 1.070(0.854,1.341) |
| CMDs | NO_X_ Q 1 | 1.909(0.880,4.140) | 1.528(1.375,1.698) | 1.167(0.991,1.373) | 1.821(1.450,2.287) |
|  | NO_X_ Q 2 | 2.541(1.313,4.917) | 1.646(1.488,1.820) | 1.265(1.085,1.476) | 2.236(1.807,2.765) |
|  | NO_X_ Q 3 | 2.566(1.360,4.843) | 1.770(1.602,1.956) | 1.340(1.151,1.561) | 2.231(1.799,2.767) |
|  | NO_X_ Q 4 | 2.997(1.650,5.443) | 1.739(1.568,1.930) | 1.319(1.126,1.545) | 2.168(1.732,2.713) |
|  | P value | 0.002 | <0.001 | 0.001 | <0.001 |

Abbreviations: PM_2.5_, particulate matter with aerodynamic diameter ≤2.5 µm; PM_10_, particulate matter with an aerodynamic diameter ≤10 µm; PM_2.5–10_, particulate matter with an aerodynamic diameter between 2.5 and 10 µm; NO_2_, nitrogen dioxide; NO_X_, nitrogen oxides; Q 1, quartile 1; CMDs, Cardiometabolic diseases; HR, hazard ratios; CI, Confidence Intervals;

In the model, we controlled for basic sociodemographic factors [age, sex, race, educational level, occupational status, TDI, BMI] and health-related concerns [APOE genotype; history of hypertension; history of depression; dyslipidemia; hypertriglyceridemia; aspirin use; lipid-lowering medication use; serum 25(OH)D levels].

Table S8. Ambient air pollution causes the incidence of mild cognitive impairment and different subtypes of dementia in patients with CMDs.

| CMDs status | Ambient air pollution | Mild cognitive impairment | | | All-cause dementia | | | Alzheimer's disease | | | Vascular dementia | | |
| --- | --- | --- | --- | --- | --- | --- | --- | --- | --- | --- | --- | --- | --- |
|  |  | Events | Person-years | per 1000 person-year | Events | Person-years | per 1000 person-year | Events | Person-years | per 1000 person-year | Events | Person-years | per 1000 person-year |
| CMDs-free | Low | 41 | 1696800 | 0.024(0.02,0.03) | 1531 | 1692362 | 0.905(0.86,0.95) | 744 | 1694893 | 0.439(0.41,0.47) | 254 | 1696392 | 0.150(0.13,0.17) |
|  | Medium | 73 | 1923399 | 0.038(0.03,0.05) | 1899 | 1918067 | 0.990(0.95,1.04) | 893 | 1921246 | 0.465(0.44,0.50) | 328 | 1922920 | 0.171(0.15,0.19) |
|  | High | 89 | 1730481 | 0.051(0.04,0.06) | 1602 | 1725895 | 0.928(0.88,0.98) | 796 | 1728652 | 0.460(0.43,0.49) | 248 | 1730345 | 0.143(0.13,0.16) |
| CMDs | Low | 14 | 339696 | 0.041(0.02,0.07) | 796 | 337517 | 2.358(2.20,2.53) | 312 | 339152 | 0.920(0.82,1.03) | 223 | 339593 | 0.657(0.57,0.75) |
|  | Medium | 26 | 436657 | 0.060(0.04,0.09) | 1160 | 433500 | 2.676(2.53,2.84) | 454 | 436047 | 1.041(0.95,1.14) | 375 | 436620 | 0.859(0.78,0.95) |
|  | High | 36 | 415437 | 0.087(0.06,0.12) | 1113 | 412363 | 2.699(2.54,2.86) | 436 | 415187 | 1.050(0.95,1.15) | 309 | 415646 | 0.743(0.66,0.83) |

Abbreviations: CMDs, Cardiometabolic disease;

Table S9. There is an association between ambient air pollutants and mild cognitive impairment and different subtypes of dementia in patients with CMDs when CMDs are used as a stratification factor.

| CMDs status | Ambient air pollution | Adjust HR (95% CI) | | | |
| --- | --- | --- | --- | --- | --- |
|  |  | Mild cognitive impairment | All-cause dementia | Alzheimer's disease | Vascular dementia |
| CMDs-free | PM_2.5_ Q 1 | 1(Ref) | 1(Ref) | 1(Ref) | 1(Ref) |
|  | PM_2.5_ Q 2 | - | 1.027(0.942,1.119) | 0.946(0.835,1.072) | - |
|  | PM_2.5_ Q 3 | - | 1.134(1.040,1.236) | 1.146(1.015,1.293) | - |
|  | PM_2.5_ Q 4 | - | 1.137(1.036,1.248) | 1.242(1.098,1.403) | - |
|  | P value | 0.316 | 0.006 | <0.001 | 0.233 |
|  | PM_2.5-10_ Q 1 | 1(Ref) | 1(Ref) | 1(Ref) | 1(Ref) |
|  | PM_2.5-10_ Q 2 | - | - | - | - |
|  | PM_2.5-10_ Q 3 | - | - | - | - |
|  | PM_2.5-10_ Q 4 | - | - | - | - |
|  | P value | 0.713 | 0.701 | 0.929 | 0.910 |
|  | PM_10_ Q 1 | 1(Ref) | 1(Ref) | 1(Ref) | 1(Ref) |
|  | PM_10_ Q 2 | - | - | - | - |
|  | PM_10_ Q 3 | - | - | - | - |
|  | PM_10_ Q 4 | - | - | - | - |
|  | P value | 0.598 | 0.326 | 0.202 | 0.367 |
|  | NO_2_ Q 1 | 1(Ref) | 1(Ref) | 1(Ref) | 1(Ref) |
|  | NO_2_ Q 2 | - | - | - | - |
|  | NO_2_ Q 3 | - | - | - | - |
|  | NO_2_ Q 4 | - | - | - | - |
|  | P value | 0.196 | 0.125 | 0.208 | 0.485 |
|  | NO_X_ Q 1 | 1(Ref) | 1(Ref) | 1(Ref) | 1(Ref) |
|  | NO_X_ Q 2 | - | - | 1.067(0.945,1.206) | - |
|  | NO_X_ Q 3 | - | - | 1.094(0.966,1.238) | - |
|  | NO_X_ Q 4 | - | - | 1.284(1.134,1.454) | - |
|  | P value | 0.179 | 0.062 | 0.001 | 0.795 |
| CMDs | PM_2.5_ Q 1 | 1(Ref) | 1(Ref) | 1(Ref) | 1(Ref) |
|  | PM_2.5_ Q 2 | - | - | - | - |
|  | PM_2.5_ Q 3 | - | - | - | - |
|  | PM_2.5_ Q 4 | - | - | - | - |
|  | P value | 0.822 | 0.245 | 0.636 | 0.586 |
|  | PM_2.5-10_ Q 1 | 1(Ref) | 1(Ref) | 1(Ref) | 1(Ref) |
|  | PM_2.5-10_ Q 2 | - | - | - | - |
|  | PM_2.5-10_ Q 3 | - | - | - | - |
|  | PM_2.5-10_ Q 4 | - | - | - | - |
|  | P value | 0.573 | 0.502 | 0.236 | 0.112 |
|  | PM_10_ Q 1 | 1(Ref) | 1(Ref) | 1(Ref) | 1(Ref) |
|  | PM_10_ Q 2 | - | 1.164(1.043,1.299) | - | - |
|  | PM_10_ Q 3 | - | 1.102(0.985,1.234) | - | - |
|  | PM_10_ Q 4 | - | 1.050(0.935,1.178) | - | - |
|  | P value | 0.912 | 0.042 | 0.327 | 0.158 |
|  | NO_2_ Q 1 | 1(Ref) | 1(Ref) | 1(Ref) | 1(Ref) |
|  | NO_2_ Q 2 | - | - | - | - |
|  | NO_2_ Q 3 | - | - | - | - |
|  | NO_2_ Q 4 | - | - | - | - |
|  | P value | 0.915 | 0.176 | 0.579 | 0.077 |
|  | NO_X_ Q 1 | 1(Ref) | 1(Ref) | 1(Ref) | 1(Ref) |
|  | NO_X_ Q 2 | - | - | - | - |
|  | NO_X_ Q 3 | - | - | - | - |
|  | NO_X_ Q 4 | - | - | - | - |
|  | P value | 0.758 | 0.105 | 0.640 | 0.153 |

Abbreviations: PM_2.5_, particulate matter with aerodynamic diameter ≤2.5 µm; PM_10_, particulate matter with an aerodynamic diameter ≤10 µm; PM_2.5–10_, particulate matter with an aerodynamic diameter between 2.5 and 10 µm; NO_2_, nitrogen dioxide; NO_X_, nitrogen oxides; Q 1, quartile 1; CMDs, Cardiometabolic diseases; HR, hazard ratios; CI, Confidence Intervals;

In the model, we controlled for basic sociodemographic factors [age, sex, race, educational level, occupational status, TDI, BMI] and health-related concerns [APOE genotype; history of hypertension; history of depression; dyslipidemia; hypertriglyceridemia; aspirin use; lipid-lowering medication use; serum 25(OH)D levels]

Table S10. Healthy lifestyle score causes the incidence of mild cognitive impairment and different subtypes of dementia in patients with CMDs.

| CMDs status | Healthy lifestyle score | Mild cognitive impairment | | | All-cause dementia rate | | | Alzheimer's disease rate | | | Vascular dementia rate | | |
| --- | --- | --- | --- | --- | --- | --- | --- | --- | --- | --- | --- | --- | --- |
|  |  | Events | Person-years | per 1000 person-year | Events | Person-years | per 1000 person-year | Events | Person-years | per 1000 person-year | Events | Person-years | per 1000 person-year |
| CMDs-free | 5-7 | 37 | 2213021 | 0.016(0.01,0.02) | 1955 | 2207164 | 0.885(0.85,0.93) | 1019 | 2210202 | 0.461(0.43,0.49) | 304 | 2212400 | 0.137(0.12,0.15) |
|  | 0-1 | 9 | 62417 | 0.144(0.07,0.28) | 83 | 62186 | 1.334(1.07,1.66) | 34 | 62379 | 0.545(0.38,0.77) | 10 | 62428 | 0.160(0.08,0.31) |
|  | 2-3 | 107 | 1480970 | 0.072(0.06,0.09) | 1469 | 1477077 | 0.994(0.94,1.05) | 656 | 1479726 | 0.443(0.41,0.48) | 250 | 1481002 | 0.168(0.15,0.19) |
|  | 4 | 50 | 1594272 | 0.031(0.02,0.04) | 1525 | 1589896 | 0.959(0.91,1.01) | 724 | 1592483 | 0.454(0.42,0.49) | 266 | 1593827 | 0.166(0.15,0.19) |
| CMDs | 0-1 | 2 | 20840 | 0.095(0.02,0.39) | 61 | 20666 | 2.951(2.28,3.82) | 19 | 20830 | 0.912(0.57,1.45) | 17 | 20862 | 0.814(0.49,1.33) |
|  | 2-3 | 37 | 404084 | 0.091(0.07,0.13) | 1149 | 401157 | 2.864(2.70,3.04) | 411 | 403687 | 1.018(0.92,1.12) | 352 | 404156 | 0.870(0.78,0.97) |
|  | 4 | 19 | 361969 | 0.052(0.03,0.09) | 897 | 359515 | 2.495(2.34,2.66) | 355 | 361543 | 0.981(0.88,1.09) | 284 | 362046 | 0.784(0.70,0.88) |
|  | 5-7 | 18 | 404897 | 0.044(0.03,0.07) | 962 | 402042 | 2.392(2.25,2.55) | 417 | 404325 | 1.031(0.94,1.14) | 254 | 404793 | 0.627(0.55,0.71) |

Abbreviations: CMDs, Cardiometabolic disease;

Table S11. There is an association between healthy lifestyle and mild cognitive impairment and different subtypes of dementia in patients with CMDs when CMDs are used as a stratification factor.

| CMDs status | Lifestyle (It is healthy?) | Adjust HR (95% CI) | | | |
| --- | --- | --- | --- | --- | --- |
|  |  | Mild cognitive impairment | All-cause dementia | Alzheimer's disease | Vascular dementia |
| CMDs-free | Diet (Yes) | - | - | - | - |
|  | P value | 0.305 | 0.060 | 0.509 | 0.557 |
|  | Smoking (Yes) | 0.290(0.203,0.415) | 0.905(0.851,0.963) | - | - |
|  | P value | <0.05 | <0.05 | 0.146 | 0.156 |
|  | Alcohol (Yes) | 0.268(0.173,0.414) | 0.925(0.868,0.985) | - | - |
|  | P value | <0.05 | <0.05 | 0.782 | 0.159 |
|  | Activity (Yes) | 0.712(0.515,0.983) | 0.846(0.792,0.903) | - | 0.811(0.690,0.954) |
|  | P value | <0.05 | <0.05 | 0.077 | <0.05 |
|  | Social (Yes) | - | 0.795(0.696,0.907) | - | - |
|  | P value | 0.159 | <0.05 | 0.393 | 0.604 |
|  | Sedentary (Yes) | - | 1.070(1.001,1.144) | - | - |
|  | P value | 0.324 | <0.05 | 0.161 | 0.45 |
|  | Sleep (Yes) | 0.714(0.520,1.127) | 0.910(0.856,0.967) | - | - |
|  | P value | <0.05 | <0.05 | 0.655 | 0.2 |
| CMDs | Diet (Yes) | - | - | - | - |
|  | P value | 0.498 | 0.599 | 0.498 | 0.744 |
|  | Smoking (Yes) | 0.386(0.220,0.679) | 0.881(0.812,0.955) | - | 0.794(0.683,0.924) |
|  | P value | <0.05 | <0.05 | 0.052 | <0.05 |
|  | Alcohol (Yes) | 0.306(0.156,0.600) | 0.875(0.806,0.949) | 0.843(0.740,0.961) | - |
|  | P value | <0.05 | <0.05 | <0.05 | 0.239 |
|  | Activity (Yes) | - | 0.887(0.818,0.962) | - | 0.799(0.690,0.924) |
|  | P value | 0.8 | <0.05 | 0.337 | <0.05 |
|  | Social (Yes) | - | - | - | - |
|  | P value | 0.932 | 0.232 | 0.093 | 0.695 |
|  | Sedentary (Yes) | - | - | - | - |
|  | P value | 0.537 | 0.34 | 0.668 | 0.104 |
|  | Sleep (Yes) | - | - | - | - |
|  | P value | 0.112 | 0.166 | 0.617 | 0.289 |

Abbreviations: CMDs, Cardiometabolic diseases; HR, hazard ratios; CI, Confidence Intervals;

In the model, we controlled for basic sociodemographic factors [age, sex, race, educational level, occupational status, TDI, BMI] and health-related concerns [APOE genotype; history of hypertension; history of depression; dyslipidemia; hypertriglyceridemia; aspirin use; lipid-lowering medication use; serum 25(OH)D levels]

Table S12. Effect modification of ambient air pollutants on mild cognitive impairment and different subtypes of dementia risk by lifestyle score in patients with CMDs when considering the combined factors of CMDs status and ambient air pollutants.

| Lifestyle | CMDs status & Ambient air pollution | Adjust HR (95% CI) | | | |
| --- | --- | --- | --- | --- | --- |
|  |  | Mild cognitive impairment | All-cause dementia | Alzheimer's disease | Vascular dementia |
| 0-1 | CMDs-free+PM_2.5_ Q1 | 1(Ref) | 1(Ref) | 1(Ref) | 1(Ref) |
|  | CMDs-free+PM_2.5_ Q2 | - | 1.892(0.744,4.811) | - | - |
|  | CMDs-free+PM_2.5_ Q3 | - | 3.047(1.294,7.174) | - | - |
|  | CMDs-free+PM_2.5_ Q4 | - | 2.532(1.089,5.889) | - | - |
|  | CMDs+PM_2.5_ Q1 | - | 1.614(0.471,5.533) | - | - |
|  | CMDs+PM_2.5_ Q2 | - | 3.490(1.364,8.933) | - | - |
|  | CMDs+PM_2.5_ Q3 | - | 2.285(0.819,6.375) | - | - |
|  | CMDs+PM_2.5_ Q4 | - | 4.913(2.105,11.463) | - | - |
|  | P value | 0.719 | 0.008 | 0.336 | 0.539 |
| 2-3 | CMDs-free+PM_2.5_ Q1 | 1(Ref) | 1(Ref) | 1(Ref) | 1(Ref) |
|  | CMDs-free+PM_2.5_ Q2 | - | 1.001(0.843,1.188) | 0.981(0.760,1.265) | 0.891(0.588,1.351) |
|  | CMDs-free+PM_2.5_ Q3 | - | 1.246(1.059,1.467) | 1.092(0.852,1.401) | 1.495(1.035,2.161) |
|  | CMDs-free+PM_2.5_ Q4 | - | 1.123(0.949,1.331) | 1.190(0.927,1.528) | 0.825(0.542,1.255) |
|  | CMDs+PM_2.5_ Q1 | - | 1.636(1.347,1.987) | 1.189(0.872,1.623) | 2.114(1.418,3.152) |
|  | CMDs+PM_2.5_ Q2 | - | 2.006(1.677,2.399) | 1.374(1.030,1.833) | 2.779(1.919,4.024) |
|  | CMDs+PM_2.5_ Q3 | - | 1.933(1.616,2.312) | 1.412(1.065,1.873) | 2.386(1.637,3.477) |
|  | CMDs+PM_2.5_ Q4 | - | 1.820(1.521,2.179) | 1.480(1.121,1.952) | 2.208(1.519,3.210) |
|  | P value | 0.055 | P<0.001 | 0.032 | P<0.001 |
| 4 | CMDs-free+PM_2.5_ Q1 | 1(Ref) | 1(Ref) | 1(Ref) | 1(Ref) |
|  | CMDs-free+PM_2.5_ Q2 | 2.501(0.888,7.042) | 1.096(0.944,1.272) | - | 1.074(0.753,1.532) |
|  | CMDs-free+PM_2.5_ Q3 | 2.099(0.730,6.041) | 1.008(0.863,1.178) | - | 0.883(0.603,1.294) |
|  | CMDs-free+PM_2.5_ Q4 | 1.254(0.402,3.907) | 1.018(0.865,1.199) | - | 0.969(0.663,1.417) |
|  | CMDs+PM_2.5_ Q1 | 5.650(1.609,19.840) | 1.400(1.160,1.689) | - | 1.893(1.307,2.743) |
|  | CMDs+PM_2.5_ Q2 | 1.005(0.116,8.710) | 1.603(1.337,1.921) | - | 2.039(1.415,2.939) |
|  | CMDs+PM_2.5_ Q3 | 4.422(1.241,15.753) | 1.558(1.300,1.867) | - | 1.979(1.376,2.846) |
|  | CMDs+PM_2.5_ Q4 | 5.872(1.803,19.122) | 1.604(1.331,1.933) | - | 1.809(1.241,2.635) |
|  | P value | 0.009 | P<0.001 | 0.348 | P<0.001 |
| 5-7 | CMDs-free+PM_2.5_ Q1 | 1(Ref) | 1(Ref) | 1(Ref) | 1(Ref) |
|  | CMDs-free+PM_2.5_ Q2 | - | 0.971(0.849,1.111) | 0.869(0.718,1.051) | 1.081(0.760,1.537) |
|  | CMDs-free+PM_2.5_ Q3 | - | 1.111(0.971,1.271) | 1.141(0.948,1.372) | 1.370(0.971,1.931) |
|  | CMDs-free+PM_2.5_ Q4 | - | 1.183(1.024,1.366) | 1.161(0.952,1.416) | 1.372(0.954,1.973) |
|  | CMDs+PM_2.5_ Q1 | - | 1.552(1.312,1.835) | 1.253(0.978,1.606) | 2.392(1.649,3.470) |
|  | CMDs+PM_2.5_ Q2 | - | 1.438(1.213,1.706) | 1.195(0.932,1.532) | 2.234(1.532,3.258) |
|  | CMDs+PM_2.5_ Q3 | - | 1.679(1.423,1.981) | 1.248(0.974,1.600) | 2.819(1.965,4.043) |
|  | CMDs+PM_2.5_ Q4 | - | 1.503(1.251,1.804) | 1.102(0.838,1.448) | 2.272(1.526,3.384) |
|  | P value | 0.140 | P<0.001 | 0.024 | P<0.001 |
| 0-1 | CMDs-free+PM_2.5-10_ Q1 | 1(Ref) | 1(Ref) | 1(Ref) | 1(Ref) |
|  | CMDs-free+PM_2.5-10_ Q 2 | - | - | - | - |
|  | CMDs-free+PM_2.5-10_ Q3 | - | - | - | - |
|  | CMDs-free+PM_2.5-10_ Q4 | - | - | - | - |
|  | CMDs+PM_2.5-10_ Q1 | - | - | - | - |
|  | CMDs+PM_2.5-10_ Q2 | - | - | - | - |
|  | CMDs+PM_2.5-10_ Q3 | - | - | - | - |
|  | CMDs+PM_2.5-10_ Q4 | - | - | - | - |
|  | P value | 0.824 | 0.468 | 0.947 | 0.772 |
| 2-3 | CMDs-free+PM_2.5-10_ Q1 | 1(Ref) | 1(Ref) | 1(Ref) | 1(Ref) |
|  | CMDs-free+PM_2.5-10_ Q 2 | - | 1.075(0.909,1.272) | 0.991(0.766,1.284) | 1.588(1.042,2.419) |
|  | CMDs-free+PM_2.5-10_ Q3 | - | 1.287(1.094,1.514) | 1.384(1.087,1.763) | 1.758(1.159,2.668) |
|  | CMDs-free+PM_2.5-10_ Q4 | - | 1.188(1.007,1.402) | 1.239(0.967,1.588) | 1.460(0.948,2.249) |
|  | CMDs+PM_2.5-10_ Q1 | - | 1.809(1.504,2.177) | 1.292(0.957,1.745) | 3.131(2.059,4.762) |
|  | CMDs+PM_2.5-10_ Q2 | - | 2.003(1.676,2.393) | 1.620(1.226,2.140) | 3.565(2.374,5.355) |
|  | CMDs+PM_2.5-10_ Q3 | - | 2.027(1.698,2.419) | 1.563(1.180,2.069) | 3.124(2.064,4.729) |
|  | CMDs+PM_2.5-10_ Q4 | - | 1.848(1.542,2.216) | 1.432(1.073,1.910) | 3.259(2.157,4.925) |
|  | P value | 0.369 | <0.001 | 0.001 | P<0.001 |
| 4 | CMDs-free+PM_2.5-10_ Q1 | 1(Ref) | 1(Ref) | 1(Ref) | 1(Ref) |
|  | CMDs-free+PM_2.5-10_ Q 2 | - | 1.052(0.907,1.220) | - | 0.661(0.451,0.968) |
|  | CMDs-free+PM_2.5-10_ Q3 | - | 1.032(0.886,1.202) | - | 1.024(0.725,1.448) |
|  | CMDs-free+PM_2.5-10_ Q4 | - | 0.878(0.749,1.030) | - | 0.752(0.516,1.097) |
|  | CMDs+PM_2.5-10_ Q1 | - | 1.483(1.241,1.772) | - | 1.792(1.264,2.542) |
|  | CMDs+PM_2.5-10_ Q2 | - | 1.578(1.322,1.883) | - | 1.981(1.405,2.794) |
|  | CMDs+PM_2.5-10_ Q3 | - | 1.381(1.145,1.666) | - | 1.463(1.006,2.128) |
|  | CMDs+PM_2.5-10_ Q4 | - | 1.477(1.228,1.776) | - | 1.478(1.016,2.152) |
|  | P value | 0.106 | P<0.001 | 0.192 | P<0.001 |
| 5-7 | CMDs-free+PM_2.5-10_ Q1 | 1(Ref) | 1(Ref) | 1(Ref) | 1(Ref) |
|  | CMDs-free+PM_2.5-10_ Q 2 | - | 0.982(0.862,1.118) | - | 1.052(0.748,1.479) |
|  | CMDs-free+PM_2.5-10_ Q3 | - | 0.856(0.746,0.982) | - | 0.802(0.550,1.168) |
|  | CMDs-free+PM_2.5-10_ Q4 | - | 0.925(0.807,1.061) | - | 1.270(0.907,1.778) |
|  | CMDs+PM_2.5-10_ Q1 | - | 1.442(1.223,1.701) | - | 1.845(1.262,2.697) |
|  | CMDs+PM_2.5-10_ Q2 | - | 1.414(1.197,1.670) | - | 2.541(1.784,3.620) |
|  | CMDs+PM_2.5-10_ Q3 | - | 1.296(1.090,1.540) | - | 1.825(1.239,2.687) |
|  | CMDs+PM_2.5-10_ Q4 | - | 1.345(1.134,1.595) | - | 2.262(1.570,3.259) |
|  | P value | 0.518 | <0.001 | 0.107 | <0.001 |
| 0-1 | CMDs-free+PM_10_ Q1 | 1(Ref) | 1(Ref) | 1(Ref) | 1(Ref) |
|  | CMDs-free+PM_10_ Q2 | - | - | - | - |
|  | CMDs-free+PM_10_ Q 3 | - | - | - | - |
|  | CMDs-free+PM_10_ Q4 | - | - | - | - |
|  | CMDs+PM_10_ Q1 | - | - | - | - |
|  | CMDs+PM_10_ Q2 | - | - | - | - |
|  | CMDs+PM_10_ Q3 | - | - | - | - |
|  | CMDs+PM_10_ Q4 | - | - | - | - |
|  | P value | 0.403 | 0.444 | 0.666 | 0.454 |
| 2-3 | CMDs-free+PM_10_ Q1 | 1(Ref) | 1(Ref) | 1(Ref) | 1(Ref) |
|  | CMDs-free+PM_10_ Q2 | - | 1.101(0.932,1.301) | 0.953(0.741,1.226) | 0.951(0.645,1.402) |
|  | CMDs-free+PM_10_ Q 3 | - | 1.230(1.043,1.451) | 1.152(0.902,1.471) | 0.923(0.621,1.373) |
|  | CMDs-free+PM_10_ Q4 | - | 1.168(0.989,1.380) | 1.133(0.887,1.448) | 1.030(0.701,1.513) |
|  | CMDs+PM_10_ Q1 | - | 1.586(1.308,1.924) | 1.108(0.815,1.506) | 1.776(1.200,2.629) |
|  | CMDs+PM_10_ Q2 | - | 2.118(1.777,2.524) | 1.309(0.985,1.739) | 2.667(1.872,3.802) |
|  | CMDs+PM_10_ Q3 | - | 1.980(1.657,2.366) | 1.736(1.333,2.262) | 1.986(1.366,2.886) |
|  | CMDs+PM_10_ Q4 | - | 1.908(1.592,2.288) | 1.255(0.938,1.680) | 2.345(1.628,3.378) |
|  | P value | 0.856 | <0.001 | 0.001 | <0.001 |
| 4 | CMDs-free+PM_10_ Q1 | 1(Ref) | 1(Ref) | 1(Ref) | 1(Ref) |
|  | CMDs-free+PM_10_ Q2 | - | 1.069(0.922,1.239) | - | 1.085(0.758,1.552) |
|  | CMDs-free+PM_10_ Q 3 | - | 0.993(0.851,1.158) | - | 0.983(0.675,1.433) |
|  | CMDs-free+PM_10_ Q4 | - | 0.868(0.739,1.020) | - | 0.901(0.611,1.327) |
|  | CMDs+PM_10_ Q1 | - | 1.393(1.159,1.675) | - | 2.074(1.437,2.991) |
|  | CMDs+PM_10_ Q2 | - | 1.521(1.275,1.815) | - | 1.952(1.352,2.819) |
|  | CMDs+PM_10_ Q3 | - | 1.606(1.341,1.923) | - | 2.069(1.427,3.001) |
|  | CMDs+PM_10_ Q4 | - | 1.363(1.128,1.646) | - | 1.716(1.166,2.526) |
|  | P value | 0.053 | <0.001 | 0.494 | <0.001 |
| 5-7 | CMDs-free+PM_10_ Q1 | 1(Ref) | 1(Ref) | 1(Ref) | 1(Ref) |
|  | CMDs-free+PM_10_ Q2 | - | 0.952(0.834,1.087) | - | 1.119(0.794,1.576) |
|  | CMDs-free+PM_10_ Q 3 | - | 0.991(0.866,1.134) | - | 0.890(0.612,1.295) |
|  | CMDs-free+PM_10_ Q4 | - | 0.933(0.812,1.072) | - | 1.345(0.957,1.891) |
|  | CMDs+PM_10_ Q1 | - | 1.428(1.207,1.688) | - | 2.080(1.428,3.028) |
|  | CMDs+PM_10_ Q2 | - | 1.520(1.288,1.794) | - | 2.565(1.788,3.678) |
|  | CMDs+PM_10_ Q3 | - | 1.325(1.115,1.574) | - | 1.956(1.331,2.873) |
|  | CMDs+PM_10_ Q4 | - | 1.387(1.167,1.648) | - | 2.328(1.605,3.376) |
|  | P value | 0.560 | <0.001 | 0.404 | <0.001 |
| 0-1 | CMDs-free+NO_2_ Q1 | 1(Ref) | 1(Ref) | 1(Ref) | 1(Ref) |
|  | CMDs-free+NO_2_ Q2 | - | - | - | 0.000(0.000,2.096^210) |
|  | CMDs-free+NO_2_ Q3 | - | - | - | 1.768(0.294,10.635) |
|  | CMDs-free+NO_2_ Q4 | - | - | - | 1.228(0.205,7.364) |
|  | CMDs+NO_2_ Q1 | - | - | - | 0.000(0.000,6.782^283) |
|  | CMDs+NO_2_ Q2 | - | - | - | 0.879(0.113,6.856) |
|  | CMDs+NO_2_ Q3 | - | - | - | 4.417(0.880,22.166) |
|  | CMDs+NO_2_ Q4 | - | - | - | 1.716(0.315,9.339) |
|  | P value | 0.851 | 0.092 | 0.496 | <0.001 |
| 2-3 | CMDs-free+NO_2_ Q1 | 1(Ref) | 1(Ref) | 1(Ref) | 1(Ref) |
|  | CMDs-free+NO_2_ Q2 | - | 1.168(0.990,1.378) | 1.033(0.806,1.323) | 1.167(0.797,1.710) |
|  | CMDs-free+NO_2_ Q3 | - | 1.095(0.925,1.296) | 0.965(0.748,1.244) | 1.076(0.728,1.588) |
|  | CMDs-free+NO_2_ Q4 | - | 1.182(0.995,1.405) | 1.227(0.953,1.581) | 0.860(0.566,1.307) |
|  | CMDs+NO_2_ Q1 | - | 1.706(1.405,2.071) | 1.229(0.905,1.671) | 2.219(1.499,3.284) |
|  | CMDs+NO_2_ Q2 | - | 1.951(1.630,2.335) | 1.374(1.035,1.823) | 2.276(1.562,3.316) |
|  | CMDs+NO_2_ Q3 | - | 1.939(1.621,2.319) | 1.416(1.072,1.870) | 2.297(1.582,3.335) |
|  | CMDs+NO_2_ Q4 | - | 1.910(1.589,2.297) | 1.385(1.039,1.847) | 2.462(1.700,3.565) |
|  | P value | 0.348 | <0.001 | 0.028 | <0.001 |
| 4 | CMDs-free+NO_2_ Q1 | 1(Ref) | 1(Ref) | 1(Ref) | 1(Ref) |
|  | CMDs-free+NO_2_ Q2 | 1.935(0.715,5.232) | 1.120(0.966,1.299) | - | 1.083(0.763,1.538) |
|  | CMDs-free+NO_2_ Q3 | 1.782(0.647,4.908) | 1.048(0.898,1.222) | - | 0.864(0.592,1.261) |
|  | CMDs-free+NO_2_ Q4 | 2.016(0.755,5.381) | 0.971(0.819,1.152) | - | 0.794(0.525,1.201) |
|  | CMDs+NO_2_ Q1 | 4.907(1.473,16.347) | 1.416(1.172,1.710) | - | 1.636(1.110,2.410) |
|  | CMDs+NO_2_ Q2 | 1.825(0.363,9.172) | 1.698(1.425,2.023) | - | 2.096(1.468,2.992) |
|  | CMDs+NO_2_ Q3 | 3.670(1.018,13.229) | 1.614(1.348,1.932) | - | 2.187(1.532,3.123) |
|  | CMDs+NO_2_ Q4 | 7.430(2.528,21.837) | 1.455(1.193,1.774) | - | 1.331(0.876,2.023) |
|  | P value | 0.010 | <0.001 | 0.536 | <0.001 |
| 5-7 | CMDs-free+NO_2_ Q1 | 1(Ref) | 1(Ref) | 1(Ref) | 1(Ref) |
|  | CMDs-free+NO_2_ Q2 | - | 1.005(0.880,1.148) | - | 1.102(0.773,1.570) |
|  | CMDs-free+NO_2_ Q3 | - | 1.132(0.991,1.294) | - | 1.407(0.997,1.985) |
|  | CMDs-free+NO_2_ Q4 | - | 1.082(0.931,1.259) | - | 1.464(1.013,2.115) |
|  | CMDs+NO_2_ Q1 | - | 1.515(1.278,1.794) | - | 2.032(1.370,3.015) |
|  | CMDs+NO_2_ Q2 | - | 1.530(1.296,1.808) | - | 2.787(1.944,3.997) |
|  | CMDs+NO_2_ Q3 | - | 1.556(1.315,1.841) | - | 2.742(1.902,3.953) |
|  | CMDs+NO_2_ Q4 | - | 1.538(1.277,1.852) | - | 2.395(1.597,3.592) |
|  | P value | 0.326 | <0.001 | 0.511 | <0.001 |
| 0-1 | CMDs-free+ NO_X_ Q1 | 1(Ref) | 1(Ref) | 1(Ref) | 1(Ref) |
|  | CMDs-free+ NO_X_ Q2 | - | 1.348(0.551,3.301) | - | - |
|  | CMDs-free+ NO_X_ Q3 | - | 2.384(1.054,5.389) | - | - |
|  | CMDs-free+ NO_X_ Q4 | - | 2.023(0.902,4.536) | - | - |
|  | CMDs+ NO_X_ Q1 | - | 0.920(0.243,3.486) | - | - |
|  | CMDs+ NO_X_ Q2 | - | 1.827(0.656,5.088) | - | - |
|  | CMDs+ NO_X_ Q3 | - | 3.076(1.289,7.341) | - | - |
|  | CMDs+ NO_X_ Q4 | - | 3.971(1.762,8.953) | - | - |
|  | P value | 0.395 | 0.006 | 0.545 | 0.787 |
| 2-3 | CMDs-free+ NO_X_ Q1 | 1(Ref) | 1(Ref) | 1(Ref) | 1(Ref) |
|  | CMDs-free+ NO_X_ Q2 | - | 1.105(0.938,1.303) | 1.032(0.806,1.320) | 0.981(0.661,1.455) |
|  | CMDs-free+ NO_X_ Q3 | - | 1.049(0.886,1.241) | 0.968(0.753,1.245) | 1.116(0.760,1.639) |
|  | CMDs-free+ NO_X_ Q4 | - | 1.130(0.954,1.338) | 1.281(1.011,1.625) | 0.934(0.625,1.396) |
|  | CMDs+ NO_X_ Q1 | - | 1.550(1.273,1.886) | 1.092(0.796,1.499) | 1.940(1.298,2.899) |
|  | CMDs+ NO_X_ Q2 | - | 1.979(1.658,2.363) | 1.408(1.063,1.865) | 2.703(1.880,3.887) |
|  | CMDs+ NO_X_ Q3 | - | 1.845(1.543,2.206) | 1.488(1.132,1.954) | 1.892(1.289,2.777) |
|  | CMDs+ NO_X_ Q4 | - | 1.847(1.543,2.210) | 1.510(1.153,1.977) | 2.499(1.739,3.591) |
|  | P value | 0.646 | <0.001 | 0.002 | <0.001 |
| 4 | CMDs-free+ NO_X_ Q1 | 1(Ref) | 1(Ref) | 1(Ref) | 1(Ref) |
|  | CMDs-free+ NO_X_ Q2 | 1.761(0.640,4.847) | 1.147(0.987,1.331) | - | 0.930(0.649,1.332) |
|  | CMDs-free+ NO_X_ Q3 | 1.425(0.494,4.110) | 1.042(0.891,1.219) | - | 0.798(0.544,1.169) |
|  | CMDs-free+ NO_X_ Q4 | 2.542(0.984,6.563) | 1.105(0.937,1.303) | - | 1.068(0.739,1.543) |
|  | CMDs+ NO_X_ Q1 | 4.986(1.496,16.621) | 1.508(1.250,1.819) | - | 1.671(1.146,2.439) |
|  | CMDs+ NO_X_ Q2 | 2.763(0.681,11.212) | 1.569(1.308,1.881) | - | 1.970(1.380,2.811) |
|  | CMDs+ NO_X_ Q3 | 1.822(0.363,9.151) | 1.736(1.451,2.078) | - | 2.087(1.466,2.970) |
|  | CMDs+ NO_X_ Q4 | 8.213(2.865,23.545) | 1.598(1.319,1.937) | - | 1.674(1.146,2.445) |
|  | P value | 0.002 | <0.001 | 0.328 | <0.001 |
| 5-7 | CMDs-free+ NO_X_ Q1 | 1(Ref) | 1(Ref) | 1(Ref) | 1(Ref) |
|  | CMDs-free+ NO_X_ Q2 | - | 1.062(0.931,1.212) | - | 1.067(0.745,1.528) |
|  | CMDs-free+ NO_X_ Q3 | - | 1.130(0.988,1.293) | - | 1.575(1.124,2.208) |
|  | CMDs-free+ NO_X_ Q4 | - | 1.073(0.924,1.247) | - | 1.390(0.959,2.015) |
|  | CMDs+ NO_X_ Q1 | - | 1.540(1.300,1.825) | - | 2.173(1.470,3.214) |
|  | CMDs+ NO_X_ Q2 | - | 1.408(1.187,1.671) | - | 2.368(1.623,3.454) |
|  | CMDs+ NO_X_ Q3 | - | 1.655(1.399,1.958) | - | 3.315(2.320,4.736) |
|  | CMDs+ NO_X_ Q4 | - | 1.635(1.366,1.958) | - | 2.768(1.876,4.085) |
|  | P value | 0.315 | <0.001 | 0.613 | <0.001 |

Abbreviations:PM_2.5_, particulate matter with aerodynamic diameter ≤2.5 µm; PM_10_, particulate matter with an aerodynamic diameter ≤10 µm; PM_2.5–10_, particulate matter with an aerodynamic diameter between 2.5 and 10 µm; NO_2_, nitrogen dioxide; NO_X_, nitrogen oxides; Q 1, quartile 1; CMDs, Cardiometabolic diseases; HR, hazard ratios; CI, Confidence Intervals;

In the model, we controlled for basic sociodemographic factors [age, sex, race, educational level, occupational status, TDI, BMI] and health-related concerns [APOE genotype; history of hypertension; history of depression; dyslipidemia; hypertriglyceridemia; aspirin use; lipid-lowering medication use; serum 25(OH)D levels]

Table S13. Effect modification of ambient air pollutants on mild cognitive impairment and different subtypes of dementia risk by lifestyle score in patients with CMDs when CMDs are used as a stratification factor.

| CMDs status | Lifestyle | Ambient air pollution | Adjust HR (95% CI) | | | |
| --- | --- | --- | --- | --- | --- | --- |
|  |  |  | Mild cognitive impairment | All-cause dementia | Alzheimer's disease | Vascular dementia |
| CMDs-free | 0-1 | PM_2.5_ Q1 | 1(Ref) | 1(Ref) | 1(Ref) | 1(Ref) |
|  |  | PM_2.5_ Q2 | - | - | - | - |
|  |  | PM_2.5_ Q3 | - | - | - | - |
|  |  | PM_2.5_ Q4 | - | - | - | - |
|  |  | P value | 0.763 | 0.056 | 0.488 | 0.654 |
|  |  | PM_2.5-10_ Q1 | 1(Ref) | 1(Ref) | 1(Ref) | 1(Ref) |
|  |  | PM_2.5-10_ Q2 | - | - | - | - |
|  |  | PM_2.5-10_ Q3 | - | - | - | - |
|  |  | PM_2.5-10_ Q4 | - | - | - | - |
|  |  | P value | 0.856 | 0.827 | 0.746 | 0.412 |
|  |  | PM_10_ Q1 | 1(Ref) | 1(Ref) | 1(Ref) | 1(Ref) |
|  |  | PM_10_ Q2 | - | - | - | - |
|  |  | PM_10_ Q3 | - | - | - | - |
|  |  | PM_10_ Q4 | - | - | - | - |
|  |  | P value | 0.268 | 0.204 | 0.193 | 0.149 |
|  |  | NO_2_ Q1 | 1(Ref) | 1(Ref) | 1(Ref) | 1(Ref) |
|  |  | NO_2_ Q2 | - | - | - | - |
|  |  | NO_2_ Q3 | - | - | - | - |
|  |  | NO_2_ Q4 | - | - | - | - |
|  |  | P value | 0.972 | 0.056 | 0.322 | 0.415 |
|  |  | NO_X_ Q1 | 1(Ref) | 1(Ref) | 1(Ref) | 1(Ref) |
|  |  | NO_X_ Q2 | - | - | - | - |
|  |  | NO_X_ Q3 | - | - | - | - |
|  |  | NO_X_ Q4 | - | - | - | - |
|  |  | P value | 0.305 | 0.123 | 0.585 | 0.965 |
|  | 2-3 | PM_2.5_ Q1 | 1(Ref) | 1(Ref) | 1(Ref) | 1(Ref) |
|  |  | PM_2.5_ Q2 | - | 1.019(0.858,1.208) | - | 0.895(0.590,1.358) |
|  |  | PM_2.5_ Q3 | - | 1.286(1.094,1.512) | - | 1.511(1.045,2.185) |
|  |  | PM_2.5_ Q4 | - | 1.222(1.038,1.439) | - | 0.837(0.549,1.276) |
|  |  | P value | 0.398 | 0.003 | 0.102 | 0.006 |
|  |  | PM_2.5-10_ Q1 | 1(Ref) | 1(Ref) | 1(Ref) | 1(Ref) |
|  |  | PM_2.5-10_ Q2 | - | 1.076(0.909,1.272) | 1.000(0.772,1.294) | - |
|  |  | PM_2.5-10_ Q3 | - | 1.292(1.097,1.521) | 1.423(1.119,1.811) | - |
|  |  | PM_2.5-10_ Q4 | - | 1.194(1.011,1.410) | 1.284(1.004,1.643) | - |
|  |  | P value | 0.884 | 0.011 | 0.005 | 0.064 |
|  |  | PM_10_ Q1 | 1(Ref) | 1(Ref) | 1(Ref) | 1(Ref) |
|  |  | PM_10_ Q2 | - | - | - | - |
|  |  | PM_10_ Q3 | - | - | - | - |
|  |  | PM_10_ Q4 | - | - | - | - |
|  |  | P value | 0.678 | 0.076 | 0.181 | 0.953 |
|  |  | NO_2_ Q1 | 1(Ref) | 1(Ref) | 1(Ref) | 1(Ref) |
|  |  | NO_2_ Q2 | - | - | 1.050(0.819,1.345) | - |
|  |  | NO_2_ Q3 | - | - | 1.000(0.777,1.286) | - |
|  |  | NO_2_ Q4 | - | - | 1.374(1.059,1.713) | - |
|  |  | P value | 0.062 | 0.154 | 0.034 | 0.466 |
|  |  | NO_X_ Q1 | 1(Ref) | 1(Ref) | 1(Ref) | 1(Ref) |
|  |  | NO_X_ Q2 | - | - | - | - |
|  |  | NO_X_ Q3 | - | - | - | - |
|  |  | NO_X_ Q4 | - | - | - | - |
|  |  | P value | 0.214 | 0.416 | 0.068 | 0.826 |
|  | 4 | PM_2.5_ Q1 | 1(Ref) | 1(Ref) | 1(Ref) | 1(Ref) |
|  |  | PM_2.5_ Q2 | - | - | - | - |
|  |  | PM_2.5_ Q3 | - | - | - | - |
|  |  | PM_2.5_ Q4 | - | - | - | - |
|  |  | P value | 0.229 | 0.575 | 0.688 | 0.833 |
|  |  | PM_2.5-10_ Q1 | 1(Ref) | 1(Ref) | 1(Ref) | 1(Ref) |
|  |  | PM_2.5-10_ Q2 | - | - | - | - |
|  |  | PM_2.5-10_ Q3 | - | - | - | - |
|  |  | PM_2.5-10_ Q4 | - | - | - | - |
|  |  | P value | 0.952 | 0.106 | 0.281 | 0.071 |
|  |  | PM_10_ Q1 | 1(Ref) | 1(Ref) | 1(Ref) | 1(Ref) |
|  |  | PM_10_ Q2 | - | - | - | - |
|  |  | PM_10_ Q3 | - | - | - | - |
|  |  | PM_10_ Q4 | - | - | - | - |
|  |  | P value | 0.552 | 0.055 | 0.248 | 0.825 |
|  |  | NO_2_ Q1 | 1(Ref) | 1(Ref) | 1(Ref) | 1(Ref) |
|  |  | NO_2_ Q2 | - | - | - | - |
|  |  | NO_2_ Q3 | - | - | - | - |
|  |  | NO_2_ Q4 | - | - | - | - |
|  |  | P value | 0.570 | 0.254 | 0.490 | 0.694 |
|  |  | NO_X_ Q1 | 1(Ref) | 1(Ref) | 1(Ref) | 1(Ref) |
|  |  | NO_X_ Q2 | - | - | - | - |
|  |  | NO_X_ Q3 | - | - | - | - |
|  |  | NO_X_ Q4 | - | - | - | - |
|  |  | P value | 0.249 | 0.352 | 0.239 | 0.516 |
|  | 5-7 | PM_2.5_ Q1 | 1(Ref) | 1(Ref) | 1(Ref) | 1(Ref) |
|  |  | PM_2.5_ Q2 | - | 0.985(0.861,1.126) | 0.891(0.737,1.078) | - |
|  |  | PM_2.5_ Q3 | - | 1.150(1.006,1.314) | 1.215(1.012,1.457) | - |
|  |  | PM_2.5_ Q4 | - | 1.275(1.111,1.464) | 1.322(1.095,1.597) | - |
|  |  | P value | 0.415 | <0.001 | <0.001 | 0.551 |
|  |  | PM_2.5-10_ Q1 | 1(Ref) | 1(Ref) | 1(Ref) | 1(Ref) |
|  |  | PM_2.5-10_ Q2 | - | - | - | - |
|  |  | PM_2.5-10_ Q3 | - | - | - | - |
|  |  | PM_2.5-10_ Q4 | - | - | - | - |
|  |  | P value | 0.847 | 0.140 | 0.178 | 0.104 |
|  |  | PM_10_ Q1 | 1(Ref) | 1(Ref) | 1(Ref) | 1(Ref) |
|  |  | PM_10_ Q2 | - | - | - | - |
|  |  | PM_10_ Q3 | - | - | - | - |
|  |  | PM_10_ Q4 | - | - | - | - |
|  |  | P value | 0.859 | 0.777 | 0.629 | 0.186 |
|  |  | NO_2_ Q1 | 1(Ref) | 1(Ref) | 1(Ref) | 1(Ref) |
|  |  | NO_2_ Q2 | - | - | - | - |
|  |  | NO_2_ Q3 | - | - | - | - |
|  |  | NO_2_ Q4 | - | - | - | - |
|  |  | P value | 0.521 | 0.171 | 0.409 | 0.516 |
|  |  | NO_X_ Q1 | 1(Ref) | 1(Ref) | 1(Ref) | 1(Ref) |
|  |  | NO_X_ Q2 | - | - | - | - |
|  |  | NO_X_ Q3 | - | - | - | - |
|  |  | NO_X_ Q4 | - | - | - | - |
|  |  | P value | 0.416 | 0.347 | 0.269 | 0.162 |
| CMDs | 0-1 | PM_2.5_ Q1 | 1(Ref) | 1(Ref) | 1(Ref) | 1(Ref) |
|  |  | PM_2.5_ Q2 | - | 2.256(0.727,7.001) | - | - |
|  |  | PM_2.5_ Q3 | - | 1502(0.452,4.995) | - | - |
|  |  | PM_2.5_ Q4 | - | 3.422(1.190,9.835) | - | - |
|  |  | P value | 0.187 | 0.046 | 0.158 | 0.272 |
|  |  | PM_2.5-10_ Q1 | 1(Ref) | 1(Ref) | 1(Ref) | 1(Ref) |
|  |  | PM_2.5-10_ Q2 | - | - | - | - |
|  |  | PM_2.5-10_ Q3 | - | - | - | - |
|  |  | PM_2.5-10_ Q4 | - | - | - | - |
|  |  | P value | 0.247 | 0.425 | 0.798 | 0.862 |
|  |  | PM_10_ Q1 | 1(Ref) | 1(Ref) | 1(Ref) | 1(Ref) |
|  |  | PM_10_ Q2 | - | - | - | - |
|  |  | PM_10_ Q3 | - | - | - | - |
|  |  | PM_10_ Q4 | - | - | - | - |
|  |  | P value | 0.247 | 0.798 | 0.966 | 0.519 |
|  |  | NO_2_ Q1 | 1(Ref) | 1(Ref) | 1(Ref) | 1(Ref) |
|  |  | NO_2_ Q2 | - | - | - | 27538.165(0.000,2.444^124) |
|  |  | NO_2_ Q3 | - | - | - | 147137.815(0.000,1.302^125) |
|  |  | NO_2_ Q4 | - | - | - | 46875.633(0.000,4.152^124) |
|  |  | P value | 0.207 | 0.094 | 0.462 | 0.126 |
|  |  | NO_X_ Q1 | 1(Ref) | 1(Ref) | 1(Ref) | 1(Ref) |
|  |  | NO_X_ Q2 | - | 2.149(0.555,8.322) | - | - |
|  |  | NO_X_ Q3 | - | 3.657(1.057,12.651) | - | - |
|  |  | NO_X_ Q4 | - | 4.644(1.399,15.410) | - | - |
|  |  | P value | 0.219 | 0.039 | 0.295 | 0.260 |
|  | 2-3 | PM_2.5_ Q1 | 1(Ref) | 1(Ref) | 1(Ref) | 1(Ref) |
|  |  | PM_2.5_ Q2 | 4.020(1.154,14.001) | - | - | - |
|  |  | PM_2.5_ Q3 | 1.069(0.239,4.786) | - | - | - |
|  |  | PM_2.5_ Q4 | 2.854(0.809,10.065) | - | - | - |
|  |  | P value | 0.034 | 0.250 | 0.752 | 0.394 |
|  |  | PM_2.5-10_ Q1 | 1(Ref) | 1(Ref) | 1(Ref) | 1(Ref) |
|  |  | PM_2.5-10_ Q2 | - | - | - | - |
|  |  | PM_2.5-10_ Q3 | - | - | - | - |
|  |  | PM_2.5-10_ Q4 | - | - | - | - |
|  |  | P value | 0.119 | 0.527 | 0.520 | 0.790 |
|  |  | PM_10_ Q1 | 1(Ref) | 1(Ref) | 1(Ref) | 1(Ref) |
|  |  | PM_10_ Q2 | - | 1.318(1.092,1.591) | 1.174(0.850,1.621) | - |
|  |  | PM_10_ Q3 | - | 1.243(1.026,1.505) | 1.546(1.137,2.103) | - |
|  |  | PM_10_ Q4 | - | 1.189(0.978,1.444) | 1.115(0.800,1.554) | - |
|  |  | P value | 0.726 | 0.033 | 0.021 | 0.106 |
|  |  | NO_2_ Q1 | 1(Ref) | 1(Ref) | 1(Ref) | 1(Ref) |
|  |  | NO_2_ Q2 | - | - | - | - |
|  |  | NO_2_ Q3 | - | - | - | - |
|  |  | NO_2_ Q4 | - | - | - | - |
|  |  | P value | 0.363 | 0.623 | 0.915 | 0.972 |
|  |  | NO_X_ Q1 | 1(Ref) | 1(Ref) | 1(Ref) | 1(Ref) |
|  |  | NO_X_ Q2 | - | - | - | - |
|  |  | NO_X_ Q3 | - | - | - | - |
|  |  | NO_X_ Q4 | - | - | - | - |
|  |  | P value | 0.307 | 0.136 | 0.495 | 0.084 |
|  | 4 | PM_2.5_ Q1 | 1(Ref) | 1(Ref) | 1(Ref) | 1(Ref) |
|  |  | PM_2.5_ Q2 | - | - | - | - |
|  |  | PM_2.5_ Q3 | - | - | - | - |
|  |  | PM_2.5_ Q4 | - | - | - | - |
|  |  | P value | 0.302 | 0.401 | 0.137 | 0.923 |
|  |  | PM_2.5-10_ Q1 | 1(Ref) | 1(Ref) | 1(Ref) | 1(Ref) |
|  |  | PM_2.5-10_ Q2 | - | - | - | - |
|  |  | PM_2.5-10_ Q3 | - | - | - | - |
|  |  | PM_2.5-10_ Q4 | - | - | - | - |
|  |  | P value | 0.947 | 0.511 | 0.173 | 0.200 |
|  |  | PM_10_ Q1 | 1(Ref) | 1(Ref) | 1(Ref) | 1(Ref) |
|  |  | PM_10_ Q2 | - | - | - | - |
|  |  | PM_10_ Q3 | - | - | - | - |
|  |  | PM_10_ Q4 | - | - | - | - |
|  |  | P value | 0.802 | 0.244 | 0.857 | 0.656 |
|  |  | NO_2_ Q1 | 1(Ref) | 1(Ref) | 1(Ref) | 1(Ref) |
|  |  | NO_2_ Q2 | - | - | - | - |
|  |  | NO_2_ Q3 | - | - | - | - |
|  |  | NO_2_ Q4 | - | - | - | - |
|  |  | P value | 0.480 | 0.143 | 0.474 | 0.084 |
|  |  | NO_X_ Q1 | 1(Ref) | 1(Ref) | 1(Ref) | 1(Ref) |
|  |  | NO_X_ Q2 | - | - | - | - |
|  |  | NO_X_ Q3 | - | - | - | - |
|  |  | NO_X_ Q4 | - | - | - | - |
|  |  | P value | 0.280 | 0.473 | 0.412 | 0.546 |
|  | 5-7 | PM_2.5_ Q1 | 1(Ref) | 1(Ref) | 1(Ref) | 1(Ref) |
|  |  | PM_2.5_ Q2 | - | - | - | - |
|  |  | PM_2.5_ Q3 | - | - | - | - |
|  |  | PM_2.5_ Q4 | - | - | - | - |
|  |  | P value | 0.132 | 0.396 | 0.403 | 0.542 |
|  |  | PM_2.5-10_ Q1 | 1(Ref) | 1(Ref) | 1(Ref) | 1(Ref) |
|  |  | PM_2.5-10_ Q2 | - | - | - | - |
|  |  | PM_2.5-10_ Q3 | - | - | - | - |
|  |  | PM_2.5-10_ Q4 | - | - | - | - |
|  |  | P value | 0.696 | 0.691 | 0.379 | 0.215 |
|  |  | PM_10_ Q1 | 1(Ref) | 1(Ref) | 1(Ref) | 1(Ref) |
|  |  | PM_10_ Q2 | - | - | - | - |
|  |  | PM_10_ Q3 | - | - | - | - |
|  |  | PM_10_ Q4 | - | - | - | - |
|  |  | P value | 0.632 | 0.514 | 0.444 | 0.492 |
|  |  | NO_2_ Q1 | 1(Ref) | 1(Ref) | 1(Ref) | 1(Ref) |
|  |  | NO_2_ Q2 | - | - | - | - |
|  |  | NO_2_ Q3 | - | - | - | - |
|  |  | NO_2_ Q4 | - | - | - | - |
|  |  | P value | 0.438 | 0.979 | 0.262 | 0.232 |
|  |  | NO_X_ Q1 | 1(Ref) | 1(Ref) | 1(Ref) | 1(Ref) |
|  |  | NO_X_ Q2 | - | - | - | - |
|  |  | NO_X_ Q3 | - | - | - | - |
|  |  | NO_X_ Q4 | - | - | - | - |
|  |  | P value | 0.419 | 0.420 | 0.816 | 0.090 |

Abbreviations:PM_2.5_, particulate matter with aerodynamic diameter ≤2.5 µm; PM_10_, particulate matter with an aerodynamic diameter ≤10 µm; PM_2.5–10_, particulate matter with an aerodynamic diameter between 2.5 and 10 µm; NO_2_, nitrogen dioxide; NO_X_, nitrogen oxides; Q 1, quartile 1; CMDs, Cardiometabolic diseases; HR, hazard ratios; CI, Confidence Intervals;

In the model, we controlled for basic sociodemographic factors [age, sex, race, educational level, occupational status, TDI, BMI] and health-related concerns [APOE genotype; history of hypertension; history of depression; dyslipidemia; hypertriglyceridemia; aspirin use; lipid-lowering medication use; serum 25(OH)D levels]

Table S14. After excluding patients with dementia in the first two years, the association between CMDs status and air pollution combined variables and mild cognitive impairment and different subtypes of dementia, and the association between air pollution and mild cognitive impairment and different subtypes of dementia in patients with CMDs when CMDs is used as a stratification factor.

| CMDs status | Ambient air pollution | Adjust HR (95%CI) | | | |
| --- | --- | --- | --- | --- | --- |
|  |  | Mild cognitive impairment | All-cause dementia | Alzheimer's disease | Vascular dementia |
| CMDs-free | Low | 1(Ref) | 1(Ref) | 1(Ref) | 1(Ref) |
|  | Medium | 1.339(0.853,2.102) | 1.101(1.022,1.186) | 1.043(0.936,1.162) | 1.159(0.965,1.391) |
|  | High | 1.230(0.770,1.965) | 1.111(1.024,1.205) | 1.166(1.038,1.309) | 0.998(0.812,1.225) |
| CMDs | Low | 2.244(1.126,4.474) | 1.539(1.398,1.693) | 1.188(1.027,1.376) | 2.019(1.648,2.473) |
|  | Medium | 2.538(1.404,4.586) | 1.767(1.620,1.927) | 1.340(1.174,1.528) | 2.582(2.147,3.105) |
|  | High | 2.600(1.460,4.628) | 1.692(1.541,1.859) | 1.275(1.105,1.471) | 2.053(1.673,2.518) |
|  | P value | 0.003 | <0.001 | <0.001 | <0.001 |
| CMDs-free | Low | 1(Ref) | 1(Ref) | 1(Ref) | 1(Ref) |
|  | Medium | - | 1.103(1.023,1.189) | 1.058(0.951,1.178) | - |
|  | High | - | 1.121(1.030,1.220) | 1.251(1.120,1.397) | - |
|  | P value | 0.397 | 0.012 | <0.001 | 0.135 |
| CMDs | Low | 1(Ref) | 1(Ref) | 1(Ref) | 1(Ref) |
|  | Medium | - | 1.140(1.033,1.259) | - | 1.290(1.078,1.544) |
|  | High | - | 1.081(0.969,1.205) | - | 1.056(0.870,1.282) |
|  | P value | 0.915 | 0.033 | 0.317 | 0.008 |

Abbreviations: CMDs, Cardiometabolic diseases; HR, hazard ratios; CI, Confidence Intervals;

* All HRs were adjusted according to age at the last follow-up, sex, race/nationality, education, occupation, body mass index (BMI), Townsend Poverty Index (TDI), APOE genotype, hypertension history, depression history, dyslipidemia, hypertriglyceridemia, aspirin usage, lipid-lowering medication usage, serum 25(OH)D levels.

Table S15. After excluding patients with dementia in the first two years, the association between CMDs status and healthy lifestyle combined variables and mild cognitive impairment and different subtypes of dementia, and the association between healthy lifestyle score and mild cognitive impairment and different subtypes of dementia in patients with CMDs when CMDs is used as a stratification factor.

| CMDs status | Healthy lifestyle score | Adjust HR(95%CI) | | | |
| --- | --- | --- | --- | --- | --- |
|  |  | Mild cognitive impairment | All-cause dementia | Alzheimer's disease | Vascular dementia |
| CMDs-free | 5-7 | 1(Ref) | 1(Ref) | 1(Ref) | 1(Ref) |
|  | 0-1 | 8.202(3.696,18.203) | 1.436(1.112,1.856) | 1.192(0.801,1.776) | 1.075(0.506,2.281) |
|  | 2-3 | 3.532(2.259,5.523) | 1.147(1.063,1.237) | 1.015(0.910,1.133) | 1.272(1.054,1.536) |
|  | 4 | 1.836(1.117,3.017) | 1.113(1.034,1.198) | 1.040(0.937,1.154) | 1.231(1.024,1.481) |
| CMDs | 0-1 | 3.069(1.523,6.183) | 1.526(1.399,1.665) | 1.179(1.037,1.340) | 2.050(1.694,2.481) |
|  | 2-3 | 3.260(0.436,24.358) | 1.994(1.497,2.655) | 1.071(0.630,1.823) | 2.653(1.538,4.574) |
|  | 4 | 6.557(3.764,11.423) | 1.909(1.753,2.079) | 1.300(1.139,1.483) | 2.743(2.284,3.295) |
|  | 5-7 | 4.331(2.273,8.254) | 1.634(1.494,1.788) | 1.175(1.025,1.347) | 2.539(2.106,3.061) |
|  | P value | <0.001 | <0.001 | 0.004 | <0.001 |
| CMDs-free | 5-7 | 1(Ref) | 1(Ref) | 1(Ref) | 1(Ref) |
|  | 0-1 | 8.057(3.613,17.969) | 1.461(1.131,1.889) | - | 1.106(0.521,2.349) |
|  | 2-3 | 3.517(2.244,5.510) | 1.156(1.072,1.248) | - | 1.290(1.067,1.559) |
|  | 4 | 1.838(1.118,3.021) | 1.116(1.037,1.201) | - | 1.239(1.030,1.490) |
|  | P value | <0.001 | <0.001 | 0.746 | 0.040 |
| CMDs | 5-7 | 1(Ref) | 1(Ref) | 1(Ref) | 1(Ref) |
|  | 0-1 | - | 1.308(0.978,1.749) | - | 1.391(0.808,2.394) |
|  | 2-3 | - | 1.251(1.137,1.377) | - | 1.399(1.169,1.674) |
|  | 4 | - | 1.073(0.972,1.186) | - | 1.265(1.051,1.521) |
|  | P value | 0.082 | <0.001 | 0.513 | 0.003 |

Abbreviations: CMDs, Cardiometabolic diseases; HR, hazard ratios; CI, Confidence Intervals;

* All HRs were adjusted according to age at the last follow-up, sex, race/nationality, education, occupation, body mass index (BMI), Townsend Poverty Index (TDI), APOE genotype, hypertension history, depression history, dyslipidemia, hypertriglyceridemia, aspirin usage, lipid-lowering medication usage, serum 25(OH)D levels.

Table S16. After excluding patients with dementia in the first two years, effect modification of CMD status and ambient air pollution combined variables on mild cognitive impairment and dementia risk by healthy lifestyle in individuals.

| Lifestyle | CMDs status & Ambient air pollution | Adjust HR(95%CI) | | | |
| --- | --- | --- | --- | --- | --- |
|  |  | Mild cognitive impairment | All-cause dementia | Alzheimer's disease | Vascular dementia |
| 0-1 | CMDs-free+ Low | 1(Ref) | 1(Ref) | 1(Ref) | 1(Ref) |
|  | CMDs-free+ Medium | - | 1.427(0.713,2.853) | - | - |
|  | CMDs-free+ High | - | 1.512(0.779,2.937) | - | - |
|  | CMDs+ Low | - | 0.964(0.342,2.716) | - | - |
|  | CMDs+ Medium | - | 2.170(1.031,4.567) | - | - |
|  | CMDs+ High | - | 3.023(1.547,5.905) | - | - |
|  | P value | 0.718 | 0.009 | 0.507 | 0.276 |
| 2-3 | CMDs-free+ Low | 1(Ref) | 1(Ref) | 1(Ref) | 1(Ref) |
|  | CMDs-free+ Medium | - | 1.088(0.941,1.259) | 0.948(0.761,1.181) | 1.308(0.936,1.826) |
|  | CMDs-free+ High | - | 1.153(0.990,1.342) | 1.265(1.023,1.564) | 0.901(0.621,1.306) |
|  | CMDs+ Low | - | 1.602(1.342,1.912) | 1.167(0.882,1.543) | 2.203(1.533,3.167) |
|  | CMDs+ Medium | - | 2.018(1.726,2.358) | 1.507(1.185,1.916) | 2.699(1.938,3.759) |
|  | CMDs+ High | - | 1.813(1.540,2.136) | 1.435(1.124,1.832) | 2.185(1.550,3.081) |
|  | P value | 0.374 | <0.001 | <0.001 | <0.001 |
| 4 | CMDs-free+ Low | 1(Ref) | 1(Ref) | 1(Ref) | 1(Ref) |
|  | CMDs-free+ Medium | - | 1.117(0.979,1.274) | - | 0.994(0.722,1.367) |
|  | CMDs-free+ High | - | 1.017(0.876,1.179) | - | 1.004(0.715,1.411) |
|  | CMDs+ Low | - | 1.462(1.232,1.735) | - | 1.832(1.298,2.585) |
|  | CMDs+ Medium | - | 1.705(1.460,1.990) | - | 2.229(1.626,3.057) |
|  | CMDs+ High | - | 1.528(1.286,1.815) | - | 1.763(1.250,2.488) |
|  | P value | 0.093 | <0.001 | 0.259 | <0.001 |
| 5-7 | CMDs-free+ Low | 1(Ref) | 1(Ref) | 1(Ref) | 1(Ref) |
|  | CMDs-free+ Medium | 0.625(0.223,1.757) | 1.090(0.969,1.225) | - | 1.300(0.962,1.757) |
|  | CMDs-free+ High | 1.540(0.647,3.664) | 1.147(1.006,1.307) | - | 1.266(0.910,1.762) |
|  | CMDs+ Low | 0.721(0.089,5.809) | 1.573(1.348,1.835) | - | 2.117(1.485,3.019) |
|  | CMDs+ Medium | 2.353(0.694,7.979) | 1.554(1.342,1.799) | - | 3.081(2.254,4.211) |
|  | CMDs+ High | 4.649(1.639,13.190) | 1.632(1.388,1.917) | - | 2.417(1.695,3.446) |
|  | P value | 0.013 | <0.001 | 0.169 | <0.001 |

Abbreviations: CMDs, Cardiometabolic diseases; HR, hazard ratios; CI, Confidence Intervals;

* All HRs were adjusted according to age at the last follow-up, sex, race/nationality, education, occupation, body mass index (BMI), Townsend Poverty Index (TDI), APOE genotype, hypertension history, depression history, dyslipidemia, hypertriglyceridemia, aspirin usage, lipid-lowering medication usage, serum 25(OH)D levels.

Table S17. After excluding patients with dementia in the first two years, effect modification of ambient air pollution on mild cognitive impairment and dementia risk by healthy lifestyle in individuals when CMDs status are used as a stratification factor.

| CMDs status | Lifestyle | Ambient air pollution | Adjust HR(95%CI) | | | |
| --- | --- | --- | --- | --- | --- | --- |
|  |  |  | Mild cognitive impairment | All-cause dementia | Alzheimer's disease | Vascular dementia |
| CMDs-free | 0-1 | Low | 1(Ref) | 1(Ref) | 1(Ref) | 1(Ref) |
|  |  | Medium | - | - | - | - |
|  |  | High | - | - | - | - |
|  |  | P value | 0.885 | 0.466 | 0.573 | 0.515 |
|  | 2-3 | Low | 1(Ref) | 1(Ref) | 1(Ref) | 1(Ref) |
|  |  | Medium | - | - | 0.948(0.761,1.181) | - |
|  |  | High | - | - | 1.270(1.026,1.571) | - |
|  |  | P value | 0.148 | 0.143 | 0.011 | 0.063 |
|  | 4 | Low | 1(Ref) | 1(Ref) | 1(Ref) | 1(Ref) |
|  |  | Medium | - | - | - | - |
|  |  | High | - | - | - | - |
|  |  | P value | 0.271 | 0.187 | 0.388 | 0.855 |
|  | 5-7 | Low | 1(Ref) | 1(Ref) | 1(Ref) | 1(Ref) |
|  |  | Medium | - | 1.112(0.990,1.250) | 1.086(0.923,1.278) | - |
|  |  | High | - | 1.229(1.085,1.392) | 1.329(1.122,1.576) | - |
|  |  | P value | 0.147 | 0.005 | 0.003 | 0.428 |
| CMDs | 0-1 | Low | 1(Ref) | 1(Ref) | 1(Ref) | 1(Ref) |
|  |  | Medium | - | 2.335(0.854,6.381) | - | - |
|  |  | High | - | 3.376(1.300,8.766) | - | - |
|  |  | P value | 0.649 | 0.037 | 0.202 | 0.108 |
|  | 2-3 | Low | 1(Ref) | 1(Ref) | 1(Ref) | 1(Ref) |
|  |  | Medium | - | 1.241(1.047,1.472) | - | - |
|  |  | High | - | 1.108(0.924,1.328) | - | - |
|  |  | P value | 0.671 | 0.037 | 0.323 | 0.223 |
|  | 4 | Low | 1(Ref) | 1(Ref) | 1(Ref) | 1(Ref) |
|  |  | Medium | - | - | - | - |
|  |  | High | - | - | - | - |
|  |  | P value | 0.745 | 0.150 | 0.197 | 0.277 |
|  | 5-7 | Low | 1(Ref) | 1(Ref) | 1(Ref) | 1(Ref) |
|  |  | Medium | - | - | - | 1.481(1.068,2.056) |
|  |  | High | - | - | - | 1.160(0.802,1.676) |
|  |  | P value | 0.072 | 0.969 | 0.286 | 0.050 |

Abbreviations: CMDs, Cardiometabolic diseases; HR, hazard ratios; CI, Confidence Intervals;

* All HRs were adjusted according to age at the last follow-up, sex, race/nationality, education, occupation, body mass index (BMI), Townsend Poverty Index (TDI), APOE genotype, hypertension history, depression history, dyslipidemia, hypertriglyceridemia, aspirin usage, lipid-lowering medication usage, serum 25(OH)D levels.

Table S18. After further adjustment for insulin resistance index, the association between CMDs status and air pollution combined variables and mild cognitive impairment and different subtypes of dementia, and the association between air pollution and mild cognitive impairment and different subtypes of dementia in patients with CMDs when CMDs is used as a stratification factor.

| CMDs status | Ambient air pollution | Adjust HR(95%CI) | | | |
| --- | --- | --- | --- | --- | --- |
|  |  | Mild cognitive impairment | All-cause dementia | Alzheimer's disease | Vascular dementia |
| CMDs-free | Low | 1(Ref) | 1(Ref) | 1(Ref) | 1(Ref) |
|  | Medium | - | 1.089(1.008,1.176) | 1.019(0.912,1.140) | 1.138(0.942,1.375) |
|  | High | - | 1.099(1.010,1.196) | 1.172(1.039,1.321) | 0.996(0.806,1.230) |
| CMDs | Low | - | 1.525(1.380,1.684) | 1.142(0.980,1.332) | 1.906(1.539,2.362) |
|  | Medium | - | 1.735(1.586,1.899) | 1.309(1.141,1.502) | 2.385(1.960,2.901) |
|  | High | - | 1.662(1.508,1.832) | 1.250(1.077,1.450) | 1.927(1.554,2.389) |
|  | P value | 0.104 | <0.001 | <0.001 | <0.001 |
| CMDs-free | Low | 1(Ref) | 1(Ref) | 1(Ref) | 1(Ref) |
|  | Medium | - | 1.091(1.010,1.179) | 1.034(0.926,1.156) | - |
|  | High | - | 1.110(1.017,1.212) | 1.250(1.116,1.401) | - |
|  | P value | 0.606 | 0.034 | <0.001 | 0.220 |
| CMDs | Low | 1(Ref) | 1(Ref) | 1(Ref) | 1(Ref) |
|  | Medium | - | - | - | 1.264(1.048,1.524) |
|  | High | - | - | - | 1.050(0.859,1.284) |
|  | P value | 0.873 | 0.077 | 0.284 | 0.023 |

Abbreviations: CMDs, Cardiometabolic diseases; HR, hazard ratios; CI, Confidence Intervals;

* All HRs were adjusted according to age at the last follow-up, sex, race/nationality, education, occupation, body mass index (BMI), Townsend Poverty Index (TDI), APOE genotype, hypertension history, depression history, dyslipidemia, hypertriglyceridemia, aspirin usage, lipid-lowering medication usage, serum 25(OH)D levels, Triglyceride-glucose index, triglyceride to high-density lipoprotein cholesterol ratio.

Table S19. After further adjustment for insulin resistance index, the association between CMDs status and healthy lifestyle combined variables and mild cognitive impairment and different subtypes of dementia, and the association between healthy lifestyle score and mild cognitive impairment and different subtypes of dementia in patients with CMDs when CMDs is used as a stratification factor.

| CMDs status | Healthy lifestyle score | Adjust HR(95%CI) | | | |
| --- | --- | --- | --- | --- | --- |
|  |  | Mild cognitive impairment | All-cause dementia | Alzheimer's disease | Vascular dementia |
| CMDs-free | 5-7 | 1(Ref) | 1(Ref) | 1(Ref) | 1(Ref) |
|  | 0-1 | 7.412(3.360,16.350) | 1.548(1.197,2.000) | 1.184(0.782,1.794) | 1.376(0.679,2.787) |
|  | 2-3 | 3.564(2.318,5.479) | 1.148(1.062,1.242) | 1.014(0.905,1.137) | 1.316(1.083,1.598) |
|  | 4 | 1.659(1.016,2.707) | 1.108(1.026,1.195) | 1.039(0.933,1.157) | 1.257(1.039,1.521) |
| CMDs | 0-1 | 2.622(1.284,5.355) | 1.504(1.374,1.647) | 1.144(1.000,1.309) | 1.949(1.592,2.385) |
|  | 2-3 | 2.862(0.384,21.333) | 1.982(1.474,2.664) | 1.208(0.722,2.020) | 2.578(1.462,4.544) |
|  | 4 | 5.523(3.175,9.606) | 1.911(1.749,2.087) | 1.279(1.115,1.468) | 2.646(2.177,3.217) |
|  | 5-7 | 4.455(2.406,8.248) | 1.618(1.474,1.776) | 1.152(0.999,1.329) | 2.467(2.024,3.007) |
|  | P value | <0.001 | <0.001 | 0.026 | <0.001 |
| CMDs-free | 5-7 | 1(Ref) | 1(Ref) | 1(Ref) | 1(Ref) |
|  | 0-1 | 7.282(3.287,16.136) | 1.576(1.219,2.038) | - | 1.366(0.674,2.769) |
|  | 2-3 | 3.517(2.282,5.419) | 1.159(1.071,1.254) | - | 1.303(1.071,1.585) |
|  | 4 | 1.654(1.013,2.699) | 1.112(1.030,1.200) | - | 1.249(1.031,1.512) |
|  | P value | <0.001 | <0.001 | 0.757 | 0.036 |
| CMDs | 5-7 | 1(Ref) | 1(Ref) | 1(Ref) | 1(Ref) |
|  | 0-1 | - | 1.322(0.979,1.785) | - | 1.326(0.754,2.331) |
|  | 2-3 | - | 1.276(1.154,1.410) | - | 1.361(1.128,1.642) |
|  | 4 | - | 1.082(0.975,1.200) | - | 1.272(1.050,1.539) |
|  | P value | 0.120 | <0.001 | 0.578 | 0.011 |

Abbreviations: CMDs, Cardiometabolic diseases; HR, hazard ratios; CI, Confidence Intervals;

* All HRs were adjusted according to age at the last follow-up, sex, race/nationality, education, occupation, body mass index (BMI), Townsend Poverty Index (TDI), APOE genotype, hypertension history, depression history, dyslipidemia, hypertriglyceridemia, aspirin usage, lipid-lowering medication usage, serum 25(OH)D levels, Triglyceride-glucose index, triglyceride to high-density lipoprotein cholesterol ratio.

Table S20. After further adjustment for insulin resistance index, effect modification of CMD status and ambient air pollution combined variables on mild cognitive impairment and dementia risk by healthy lifestyle in individuals.

| Lifestyle | CMDs status & Ambient air pollution | Adjust HR(95%CI) | | | |
| --- | --- | --- | --- | --- | --- |
|  |  | Mild cognitive impairment | All-cause dementia | Alzheimer's disease | Vascular dementia |
| 0-1 | CMDs-free+ Low | 1(Ref) | 1(Ref) | 1(Ref) | 1(Ref) |
|  | CMDs-free+ Medium | - | - | - | - |
|  | CMDs-free+ High | - | - | - | - |
|  | CMDs+ Low | - | - | - | - |
|  | CMDs+ Medium | - | - | - | - |
|  | CMDs+ High | - | - | - | - |
|  | P value | 0.719 | 0.195 | 0.319 | 0.273 |
| 2-3 | CMDs-free+ Low | 1(Ref) | 1(Ref) | 1(Ref) | 1(Ref) |
|  | CMDs-free+ Medium | - | 1.093(0.940,1.271) | 0.918(0.730,1.154) | 1.418(1.001,2.010) |
|  | CMDs-free+ High | - | 1.131(0.966,1.324) | 1.271(1.021,1.582) | 0.938(0.636,1.382) |
|  | CMDs+ Low | - | 1.668(1.391,2.001) | 1.163(0.872,1.551) | 2.080(1.408,3.074) |
|  | CMDs+ Medium | - | 1.979(1.682,2.328) | 1.378(1.070,1.775) | 2.572(1.798,3.679) |
|  | CMDs+ High | - | 1.788(1.509,2.119) | 1.360(1.055,1.754) | 2.095(1.448,3.031) |
|  | P value | 0.696 | <0.001 | 0.003 | <0.001 |
| 4 | CMDs-free+ Low | 1(Ref) | 1(Ref) | 1(Ref) | 1(Ref) |
|  | CMDs-free+ Medium | 2.000(0.822,4.865) | 1.075(0.938,1.233) | - | 0.956(0.688,1.330) |
|  | CMDs-free+ High | 1.652(0.657,4.151) | 0.981(0.841,1.144) | - | 1.006(0.709,1.426) |
|  | CMDs+ Low | 4.846(1.495,15.705) | 1.431(1.198,1.709) | - | 1.770(1.241,2.524) |
|  | CMDs+ Medium | 3.128(0.893,10.961) | 1.639(1.396,1.924) | - | 2.065(1.488,2.865) |
|  | CMDs+ High | 7.182(2.600,19.840) | 1.461(1.223,1.746) | - | 1.754(1.234,2.492) |
|  | P value | 0.002 | <0.001 | 0.550 | <0.001 |
| 5-7 | CMDs-free+ Low | 1(Ref) | 1(Ref) | 1(Ref) | 1(Ref) |
|  | CMDs-free+ Medium | - | 1.085(0.961,1.225) | - | 1.184(0.867,1.617) |
|  | CMDs-free+ High | - | 1.153(1.007,1.321) | - | 1.219(0.869,1.710) |
|  | CMDs+ Low | - | 1.521(1.294,1.788) | - | 1.934(1.335,2.801) |
|  | CMDs+ Medium | - | 1.570(1.349,1.827) | - | 2.707(1.946,3.765) |
|  | CMDs+ High | - | 1.609(1.359,1.904) | - | 2.092(1.440,3.041) |
|  | P value | 0.095 | <0.001 | 0.166 | <0.001 |

Abbreviations: CMDs, Cardiometabolic diseases; HR, hazard ratios; CI, Confidence Intervals;

* All HRs were adjusted according to age at the last follow-up, sex, race/nationality, education, occupation, body mass index (BMI), Townsend Poverty Index (TDI), APOE genotype, hypertension history, depression history, dyslipidemia, hypertriglyceridemia, aspirin usage, lipid-lowering medication usage, serum 25(OH)D levels, Triglyceride-glucose index, triglyceride to high-density lipoprotein cholesterol ratio.

Table S21. After further adjustment for insulin resistance index, effect modification of ambient air pollution on mild cognitive impairment and dementia risk by healthy lifestyle in individuals when CMDs status are used as a stratification factor.

| CMDs status | Lifestyle | Ambient air pollution | Adjust HR(95%CI) | | | |
| --- | --- | --- | --- | --- | --- | --- |
|  |  |  | Mild cognitive impairment | All-cause dementia | Alzheimer's disease | Vascular dementia |
| CMDs-free | 0-1 | Low | 1(Ref) | 1(Ref) | 1(Ref) | 1(Ref) |
|  |  | Medium | - | - | - | - |
|  |  | High | - | - | - | - |
|  |  | P value | 0.886 | 0.334 | 0.634 | 0.818 |
|  | 2-3 | Low | 1(Ref) | 1(Ref) | 1(Ref) | 1(Ref) |
|  |  | Medium | - | - | 0.936(0.752,1.165) | - |
|  |  | High | - | - | 1.267(1.025,1.566) | - |
|  |  | P value | 0.191 | 0.159 | 0.008 | 0.064 |
|  | 4 | Low | 1(Ref) | 1(Ref) | 1(Ref) | 1(Ref) |
|  |  | Medium | - | - | - | - |
|  |  | High | - | - | - | - |
|  |  | P value | 0.228 | 0.288 | 0.345 | 0.999 |
|  | 5-7 | Low | 1(Ref) | 1(Ref) | 1(Ref) | 1(Ref) |
|  |  | Medium | - | 1.115(0.993,1.251) | 1.084(0.922,1.274) | - |
|  |  | High | - | 1.235(1.091,1.397) | 1.328(1.123,1.572) | - |
|  |  | P value | 0.532 | 0.004 | 0.003 | 0.393 |
| CMDs | 0-1 | Low | 1(Ref) | 1(Ref) | 1(Ref) | 1(Ref) |
|  |  | Medium | - | 2.320(0.849,6.340) | - | - |
|  |  | High | - | 3.488(1.347,9.036) | - | - |
|  |  | P value | 0.159 | 0.028 | 0.141 | 0.108 |
|  | 2-3 | Low | 1(Ref) | 1(Ref) | 1(Ref) | 1(Ref) |
|  |  | Medium | - | - | - | - |
|  |  | High | - | - | - | - |
|  |  | P value | 0.573 | 0.103 | 0.442 | 0.326 |
|  | 4 | Low | 1(Ref) | 1(Ref) | 1(Ref) | 1(Ref) |
|  |  | Medium | - | - | - | - |
|  |  | High | - | - | - | - |
|  |  | P value | 0.474 | 0.166 | 0.170 | 0.362 |
|  | 5-7 | Low | 1(Ref) | 1(Ref) | 1(Ref) | 1(Ref) |
|  |  | Medium | - | - | - | 1.497(1.079,2.076) |
|  |  | High | - | - | - | 1.202(0.834,1.732) |
|  |  | P value | 0.100 | 0.948 | 0.258 | 0.047 |

Abbreviations: CMDs, Cardiometabolic diseases; HR, hazard ratios; CI, Confidence Intervals;

* All HRs were adjusted according to age at the last follow-up, sex, race/nationality, education, occupation, body mass index (BMI), Townsend Poverty Index (TDI), APOE genotype, hypertension history, depression history, dyslipidemia, hypertriglyceridemia, aspirin usage, lipid-lowering medication usage, serum 25(OH)D levels, Triglyceride-glucose index, triglyceride to high-density lipoprotein cholesterol ratio.

Table S22. After removing missing values of covariates, the association between CMDs status and air pollution combined variables and mild cognitive impairment and different subtypes of dementia, and the association between air pollution and mild cognitive impairment and different subtypes of dementia in patients with CMDs when CMDs is used as a stratification factor.

| CMDs status | Ambient air pollution | Adjust HR(95%CI) | | | |
| --- | --- | --- | --- | --- | --- |
|  |  | Mild cognitive impairment | All-cause dementia | Alzheimer's disease | Vascular dementia |
| CMDs-free | Low | 1(Ref) | 1(Ref) | 1(Ref) | 1(Ref) |
|  | Medium | 1.363(0.888,2.091) | 1.095(1.017,1.179) | 1.040(0.934,1.157) | 1.162(0.969,1.394) |
|  | High | 1.290(0.830,2.006) | 1.109(1.022,1.202) | 1.163(1.036,1.305) | 0.999(0.815,1.225) |
| CMDs | Low | 2.117(1.093,4.102) | 1.541(1.402,1.694) | 1.186(1.025,1.372) | 1.939(1.579,2.381) |
|  | Medium | 2.485(1.421,4.344) | 1.740(1.596,1.897) | 1.325(1.162,1.511) | 2.455(2.034,2.961) |
|  | High | 2.562(1.487,4.413) | 1.686(1.536,1.851) | 1.267(1.099,1.461) | 2.006(1.633,2.465) |
|  | P value | 0.003 | <0.001 | <0.001 | <0.001 |
| CMDs-free | Low | 1(Ref) | 1(Ref) | 1(Ref) | 1(Ref) |
|  | Medium | - | 1.097(1.018,1.182) | 1.057(0.950,1.175) | - |
|  | High | - | 1.120(1.030,1.218) | 1.253(1.123,1.398) | - |
|  | P value | 0.339 | 0.014 | <0.001 | 0.127 |
| CMDs | Low | 1(Ref) | 1(Ref) | 1(Ref) | 1(Ref) |
|  | Medium | - | - | - | 1.283(1.073,1.535) |
|  | High | - | - | - | 1.086(0.897,1.315) |
|  | P value | 0.829 | 0.074 | 0.372 | 0.015 |

Abbreviations: CMDs, Cardiometabolic diseases; HR, hazard ratios; CI, Confidence Intervals;

* All HRs were adjusted according to age at the last follow-up, sex, race/nationality, education, occupation, body mass index (BMI), Townsend Poverty Index (TDI), APOE genotype, hypertension history, depression history, dyslipidemia, hypertriglyceridemia, aspirin usage, lipid-lowering medication usage, serum 25(OH)D levels.

Table S23. After removing missing values of covariates, the association between CMDs status and healthy lifestyle combined variables and mild cognitive impairment and different subtypes of dementia, and the association between healthy lifestyle score and mild cognitive impairment and different subtypes of dementia in patients with CMDs when CMDs is used as a stratification factor.

| CMDs status | Healthy lifestyle score | Adjust HR(95%CI) | | | |
| --- | --- | --- | --- | --- | --- |
|  |  | Mild cognitive impairment | All-cause dementia | Alzheimer's disease | Vascular dementia |
| CMDs-free | 5-7 | 1(Ref) | 1(Ref) | 1(Ref) | 1(Ref) |
|  | 0-1 | 6.478(2.949,14.233) | 1.478(1.151,1.899) | 1.219(0.825,1.802) | 1.180(0.583,2.388) |
|  | 2-3 | 3.523(2.324,5.340) | 1.141(1.058,1.230) | 1.007(0.903,1.123) | 1.227(1.018,1.479) |
|  | 4 | 1.655(1.032,2.655) | 1.110(1.032,1.193) | 1.037(0.935,1.150) | 1.207(1.006,1.449) |
| CMDs | 0-1 | 2.973(1.550,5.703) | 1.516(1.391,1.653) | 1.169(1.029,1.328) | 1.940(1.600,2.352) |
|  | 2-3 | 2.422(0.326,18.010) | 1.990(1.498,2.643) | 1.128(0.675,1.885) | 2.447(1.417,4.225) |
|  | 4 | 5.596(3.307,9.469) | 1.901(1.746,2.068) | 1.287(1.129,1.468) | 2.592(2.153,3.120) |
|  | 5-7 | 4.162(2.290,7.565) | 1.622(1.484,1.773) | 1.167(1.018,1.336) | 2.394(1.982,2.893) |
|  | P value | <0.001 | <0.001 | 0.005 | <0.001 |
| CMDs-free | 5-7 | 1(Ref) | 1(Ref) | 1(Ref) | 1(Ref) |
|  | 0-1 | 6.152(2.786,13.587) | 1.504(1.170,1.932) | - | - |
|  | 2-3 | 3.421(2.251,5.199) | 1.150(1.066,1.240) | - | - |
|  | 4 | 1.639(1.021,2.629) | 1.113(1.034,1.197) | - | - |
|  | P value | <0.001 | <0.001 | 0.691 | 0.091 |
| CMDs | 5-7 | 1(Ref) | 1(Ref) | 1(Ref) | 1(Ref) |
|  | 0-1 | - | 1.314(0.985,1.753) | - | 1.354(0.787,2.330) |
|  | 2-3 | - | 1.253(1.139,1.379) | - | 1.391(1.165,1.662) |
|  | 4 | - | 1.072(0.971,1.184) | - | 1.256(1.046,1.509) |
|  | P value | 0.108 | <0.001 | 0.566 | 0.003 |

Abbreviations: CMDs, Cardiometabolic diseases; HR, hazard ratios; CI, Confidence Intervals;

* All HRs were adjusted according to age at the last follow-up, sex, race/nationality, education, occupation, body mass index (BMI), Townsend Poverty Index (TDI), APOE genotype, hypertension history, depression history, dyslipidemia, hypertriglyceridemia, aspirin usage, lipid-lowering medication usage, serum 25(OH)D levels.

Table S24. After removing missing values of covariates, effect modification of CMD status and ambient air pollution combined variables on mild cognitive impairment and dementia risk by healthy lifestyle in individuals.

| Lifestyle | CMDs status & Ambient air pollution | Adjust HR(95%CI) | | | |
| --- | --- | --- | --- | --- | --- |
|  |  | Mild cognitive impairment | All-cause dementia | Alzheimer's disease | Vascular dementia |
| 0-1 | CMDs-free+ Low | 1(Ref) | 1(Ref) | 1(Ref) | 1(Ref) |
|  | CMDs-free+ Medium | - | 1.521(0.765,3.023) | - | - |
|  | CMDs-free+ High | - | 1.672(0.867,3.227) | - | - |
|  | CMDs+ Low | - | 0.936(0.332,2.638) | - | - |
|  | CMDs+ Medium | - | 2.191(1.040,4.616) | - | - |
|  | CMDs+ High | - | 3.049(1.559,5.965) | - | - |
|  | P value | 0.719 | 0.010 | 0.416 | 0.417 |
| 2-3 | CMDs-free+ Low | 1(Ref) | 1(Ref) | 1(Ref) | 1(Ref) |
|  | CMDs-free+ Medium | - | 1.083(0.937,1.252) | 0.936(0.752,1.165) | 1.308(0.937,1.828) |
|  | CMDs-free+ High | - | 1.144(0.984,1.330) | 1.263(1.023,1.560) | 0.901(0.621,1.307) |
|  | CMDs+ Low | - | 1.645(1.382,1.958) | 1.179(0.894,1.554) | 2.279(1.589,3.268) |
|  | CMDs+ Medium | - | 1.986(1.701,2.320) | 1.476(1.161,1.876) | 2.717(1.952,3.782) |
|  | CMDs+ High | - | 1.805(1.534,2.124) | 1.432(1.123,1.825) | 2.270(1.614,3.194) |
|  | P value | 0.505 | <0.001 | 0.003 | <0.001 |
| 4 | CMDs-free+ Low | 1(Ref) | 1(Ref) | 1(Ref) | 1(Ref) |
|  | CMDs-free+ Medium | 2.170(0.900,5.235) | 1.098(0.963,1.252) | - | 0.978(0.713,1.341) |
|  | CMDs-free+ High | 2.003(0.815,4.922) | 1.011(0.873,1.171) | - | 0.975(0.695,1.367) |
|  | CMDs+ Low | 4.132(1.289,13.243) | 1.438(1.213,1.705) | - | 1.788(1.270,2.518) |
|  | CMDs+ Medium | 2.694(0.775,9.359) | 1.673(1.435,1.952) | - | 2.171(1.587,2.969) |
|  | CMDs+ High | 7.110(2.649,19.083) | 1.525(1.286,1.808) | - | 1.789(1.275,2.510) |
|  | P value | 0.003 | <0.001 | 0.236 | <0.001 |
| 5-7 | CMDs-free+ Low | 1(Ref) | 1(Ref) | 1(Ref) | 1(Ref) |
|  | CMDs-free+ Medium | - | 1.091(0.972,1.226) | - | 1.324(0.983,1.784) |
|  | CMDs-free+ High | - | 1.150(1.010,1.309) | - | 1.326(0.959,1.835) |
|  | CMDs+ Low | - | 1.565(1.343,1.824) | - | 1.945(1.355,2.793) |
|  | CMDs+ Medium | - | 1.538(1.329,1.779) | - | 2.872(2.089,3.950) |
|  | CMDs+ High | - | 1.623(1.383,1.906) | - | 2.315(1.620,3.308) |
|  | P value | 0.161 | <0.001 | 0.179 | <0.001 |

Abbreviations: CMDs, Cardiometabolic diseases; HR, hazard ratios; CI, Confidence Intervals;

* All HRs were adjusted according to age at the last follow-up, sex, race/nationality, education, occupation, body mass index (BMI), Townsend Poverty Index (TDI), APOE genotype, hypertension history, depression history, dyslipidemia, hypertriglyceridemia, aspirin usage, lipid-lowering medication usage, serum 25(OH)D levels.

Table S25. After removing missing values of covariates, effect modification of ambient air pollution on mild cognitive impairment and dementia risk by healthy lifestyle in individuals when CMDs status are used as a stratification factor.

| CMDs status | Lifestyle | Ambient air pollution | Adjust HR(95%CI) | | | |
| --- | --- | --- | --- | --- | --- | --- |
|  |  |  | Mild cognitive impairment | All-cause dementia | Alzheimer's disease | Vascular dementia |
| CMDs-free | 0-1 | Low | 1(Ref) | 1(Ref) | 1(Ref) | 1(Ref) |
|  |  | Medium | - | - | - | - |
|  |  | High | - | - | - | - |
|  |  | P value | 0.886 | 0.334 | 0.634 | 0.818 |
|  | 2-3 | Low | 1(Ref) | 1(Ref) | 1(Ref) | 1(Ref) |
|  |  | Medium | - | - | 0.936(0.752,1.165) | - |
|  |  | High | - | - | 1.267(1.025,1.566) | - |
|  |  | P value | 0.191 | 0.159 | 0.008 | 0.064 |
|  | 4 | Low | 1(Ref) | 1(Ref) | 1(Ref) | 1(Ref) |
|  |  | Medium | - | - | - | - |
|  |  | High | - | - | - | - |
|  |  | P value | 0.228 | 0.288 | 0.345 | 0.999 |
|  | 5-7 | Low | 1(Ref) | 1(Ref) | 1(Ref) | 1(Ref) |
|  |  | Medium | - | 1.115(0.993,1.251) | 1.084(0.922,1.278) | - |
|  |  | High | - | 1.235(1.091,1.397) | 1.328(1.123,1.572) | - |
|  |  | P value | 0.532 | 0.004 | 0.003 | 0.393 |
| CMDs | 0-1 | Low | 1(Ref) | 1(Ref) | 1(Ref) | 1(Ref) |
|  |  | Medium | - | 2.320(0.849,6.340) | - | - |
|  |  | High | - | 3.488(1.347,9.036) | - | - |
|  |  | P value | 0.159 | 0.028 | 0.141 | 0.108 |
|  | 2-3 | Low | 1(Ref) | 1(Ref) | 1(Ref) | 1(Ref) |
|  |  | Medium | - | - | - | - |
|  |  | High | - | - | - | - |
|  |  | P value | 0.573 | 0.103 | 0.442 | 0.326 |
|  | 4 | Low | 1(Ref) | 1(Ref) | 1(Ref) | 1(Ref) |
|  |  | Medium | - | - | - | - |
|  |  | High | - | - | - | - |
|  |  | P value | 0.474 | 0.166 | 0.170 | 0.362 |
|  | 5-7 | Low | 1(Ref) | 1(Ref) | 1(Ref) | 1(Ref) |
|  |  | Medium | - | - | - | 1.497(1.079,2.076) |
|  |  | High | - | - | - | 1.202(0.834,1.732) |
|  |  | P value | 0.100 | 0.948 | 0.258 | 0.047 |

Abbreviations: CMDs, Cardiometabolic diseases; HR, hazard ratios; CI, Confidence Intervals;

* All HRs were adjusted according to age at the last follow-up, sex, race/nationality, education, occupation, body mass index (BMI), Townsend Poverty Index (TDI), APOE genotype, hypertension history, depression history, dyslipidemia, hypertriglyceridemia, aspirin usage, lipid-lowering medication usage, serum 25(OH)D levels.

Figure S1. Flowchart of participants included in the analysis


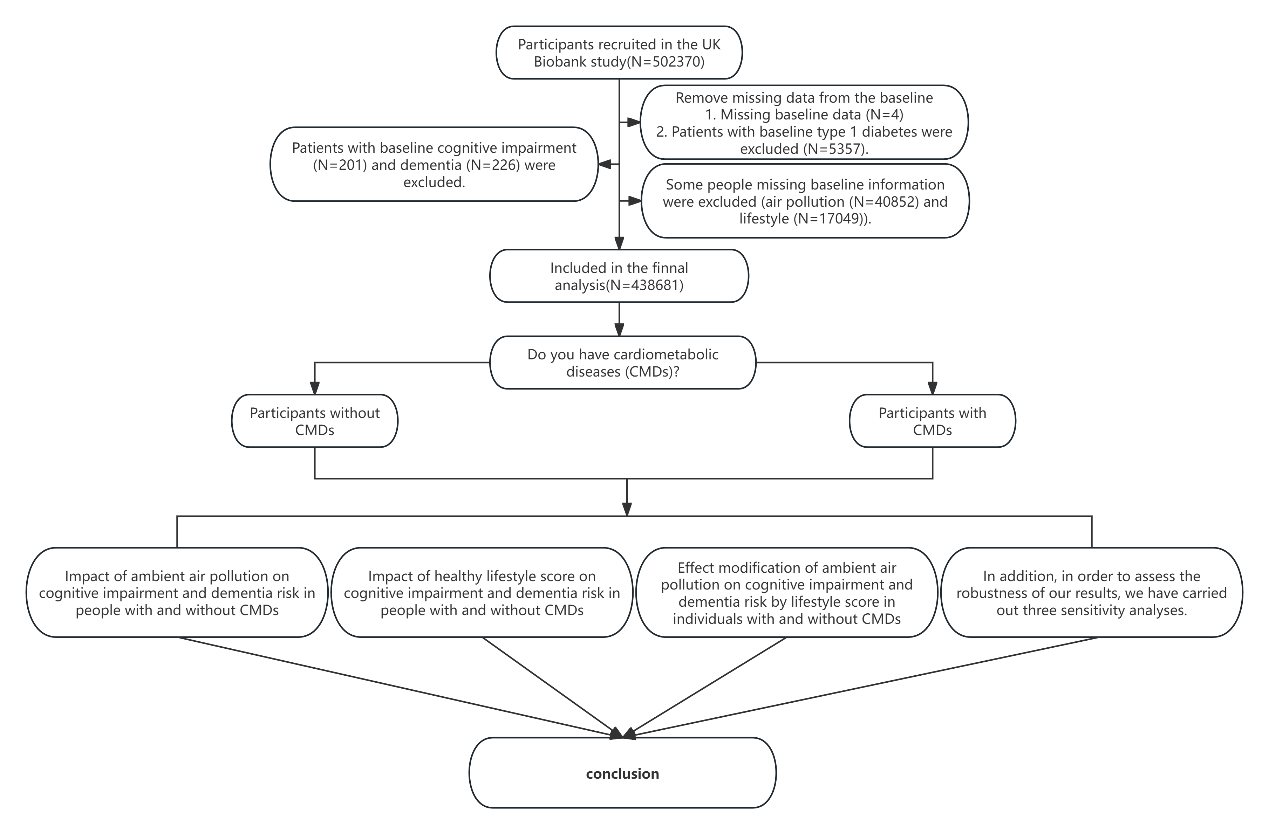


Figure S2. Category difference of comprehensive variables of ambient air pollution


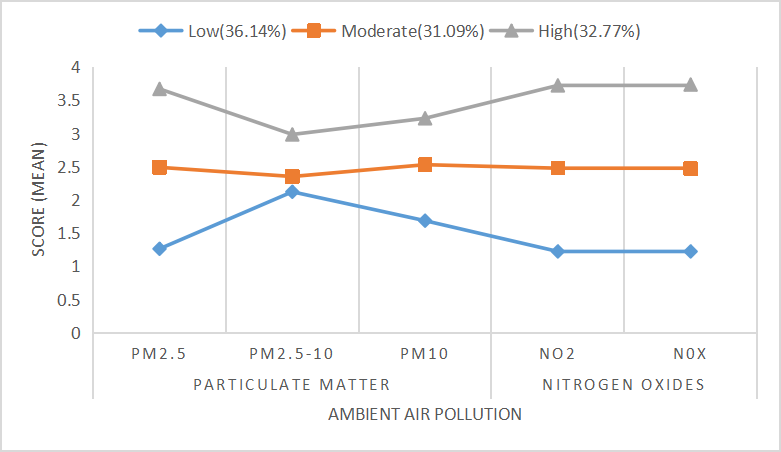

Supplement: Supplementary file 1 — Supplementary Material 1 [file 41598_2024_83607_MOESM1_ESM.docx]
